# Spatio-dipolar synergy modulated interfacial molecular bridge for calendar-aging-resistant aqueous zinc-ion batteries

**DOI:** 10.1093/nsr/nwag268

**Published:** 2026-05-09

**Authors:** Zimin Yang, Yilun Sun, Jianwei Li, Mingqiang Wu, Siting Deng, Xinbin Nie, Yifan Su, Hao Tong, Pengpeng Guo, Jingkui Gao, Guoliang Chai

**Affiliations:** State Key Laboratory of Structural Chemistry, Fujian Institute of Research on the Structure of Matter, Chinese Academy of Sciences, Fuzhou 350002, China; Fujian College, University of Chinese Academy of Sciences, Fuzhou 350002, China; School of Chemical Science, University of Chinese Academy of Sciences, Beijing 100049, China; State Key Laboratory of Structural Chemistry, Fujian Institute of Research on the Structure of Matter, Chinese Academy of Sciences, Fuzhou 350002, China; Key Laboratory of Comprehensive and Highly Efficient Utilization of Salt Lake Resources, Qinghai Province Key Laboratory of Resources and Chemistry of Salt Lakes, Qinghai Institute of Salt Lakes, Chinese Academy of Sciences, Xining 810008, China; School of Chemical Science, University of Chinese Academy of Sciences, Beijing 100049, China; State Key Laboratory of Structural Chemistry, Fujian Institute of Research on the Structure of Matter, Chinese Academy of Sciences, Fuzhou 350002, China; College of Chemistry, Fuzhou University, Fuzhou 350108, China; State Key Laboratory of Structural Chemistry, Fujian Institute of Research on the Structure of Matter, Chinese Academy of Sciences, Fuzhou 350002, China; Key Laboratory of Comprehensive and Highly Efficient Utilization of Salt Lake Resources, Qinghai Province Key Laboratory of Resources and Chemistry of Salt Lakes, Qinghai Institute of Salt Lakes, Chinese Academy of Sciences, Xining 810008, China; Key Laboratory of Comprehensive and Highly Efficient Utilization of Salt Lake Resources, Qinghai Province Key Laboratory of Resources and Chemistry of Salt Lakes, Qinghai Institute of Salt Lakes, Chinese Academy of Sciences, Xining 810008, China; State Key Laboratory of Structural Chemistry, Fujian Institute of Research on the Structure of Matter, Chinese Academy of Sciences, Fuzhou 350002, China; College of Chemistry, Fuzhou University, Fuzhou 350108, China; College of Chemistry, Fuzhou University, Fuzhou 350108, China; State Key Laboratory of Structural Chemistry, Fujian Institute of Research on the Structure of Matter, Chinese Academy of Sciences, Fuzhou 350002, China; Fujian College, University of Chinese Academy of Sciences, Fuzhou 350002, China; School of Chemical Science, University of Chinese Academy of Sciences, Beijing 100049, China

**Keywords:** aqueous Zn-ion batteries, additive, calendar aging, spatio-dipolar synergy

## Abstract

Short calendar-aging life and interfacial degradation hinder the applications of aqueous Zn-ion batteries. Herein, trimorpholinophosphine oxide (TPO), featuring a high topological polar surface area (TPSA), is introduced as an electrolyte additive that modulates interfacial ions via steric-dipolar synergism. Specifically, the topological polarity of TPO facilitates Zn^2+^ coordination and strong surface adsorption, creating migration channels within solvation-interfacial networks. Moreover, the steric hindrance of TPO disrupts the solvation sheath of Zn^2+^, forming H_3_O^+^-repelled layers. Consequently, TPO-containing cells achieve an exceptional average Coulombic efficiency (CE) of 99.91% and operate steadily at ultrahigh current densities (280 mA cm^−2^). At −20°C, TPO-containing cells retain 177 mAh g⁻^1^ after 6000 cycles, yet additive-free cells fail after 1400 cycles (84 mAh g⁻^1^). Most importantly, TPO-containing cells maintain a high CE exceeding 95% for over 2500 h under intermittent calendar-aging, far exceeding the 72 h of additive-free cells. This work offers molecular-level insights into TPSA-induced steric-dipole synergism for ultra-stable batteries.

## Teaser

Guiding steric-dipolar synergy with topological polar surface area (TPSA): A rationally designed electrolyte additive suppresses anode aging, granting aqueous zinc-ion batteries an exceptionally long calendar life.

## INTRODUCTION

Aqueous zinc-ion batteries (AZIBs) have attracted considerable attention as promising candidates for sustainable energy storage, owing to their non-flammability, cost-effectiveness, and environmental compatibility [1–4]. Metallic zinc anodes possess inherent merits including high theoretical capacity (820 mAh g^−1^) and a low redox potential (−0.76 V vs. SHE) [5–7], yet their applications in AZIBs remain hindered by persistent challenges: anode corrosion, dendritic growth, irreversible byproduct formation, and narrow operational temperature range [8–11]. Of particular concern is the spontaneous corrosion behavior involving parasitic hydrogen evolution reaction, which persists throughout battery operation and becomes particularly severe during prolonged battery aging periods. In practical energy storage applications, batteries inevitably undergo prolonged calendar aging under intermittent charge/discharge cycling [5]. This critical precondition has been frequently overlooked in previous investigations of AZIBs [12–14]. Calendar-aging life serves as a critical parameter for evaluating long-term stability in energy storage systems [15]. It quantifies irreversible degradation mechanisms under real-storage conditions [12,16,17]. To date, only a limited number of studies have delved into this significant aspect [5,10,12–16,18–20], limiting the translation of laboratory-scale advancements into commercially viable energy storage solutions.

Notably, the repulsion of interfacial active H_2_O and H_3_O^+^ can effectively suppress interfacial parasitic reactions and reduce the desolvation energy barrier, thereby facilitating the ordered migration of Zn^2+^ toward the electrode interface [9]. Therefore, achieving interfacial ions (H_3_O^+^, Zn^2+^) redistribution and optimizing the interfacial framework is promising to improve the calendar aging and high-rate performance for AZIBs. Electrolyte additive engineering [21–25], such as organic co-solvents [26], or highly concentrated electrolytes [27], is a feasible strategy to modify the electrolyte-electrode interface and hence optimize interfacial ions (H_3_O^+^, Zn^2+^) redistribution [28]. Specifically, the additives with steric hindrance effects are capable of tuning the solvation sheath of Zn^2+^ for forming a water-deficient layer via excluding water molecules. Furthermore, ion/dipole-dipole effects can enhance the interaction between additive and Zn^2+^, thereby improving Zn^2+^ migration kinetics [29]. However, how to design additives with steric hindrance and efficient ion/dipole-dipole effect synergism is still a challenge. This synergy refers to the complementary combination of steric hindrance, which physically excludes interfacial H_3_O^+^ and displaces water from the Zn^2+^ solvation sheath, and spatially distributed ion/dipole interactions, which enable strong surface adsorption and facilitate Zn^2+^ coordination. The design challenge lies in balancing these two functionalities, as steric groups may compromise the accessibility of polar coordination sites, while purely polar additives often lack sufficient water-shielding capability.

**Figure 1.**
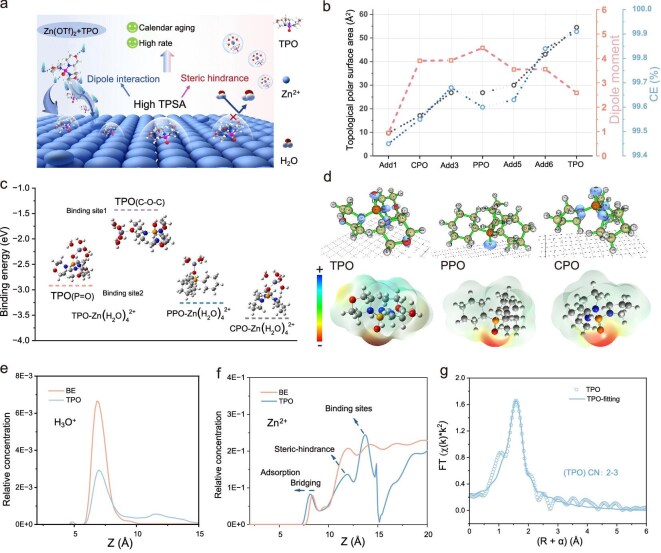
Additive screening and topological modulation of solution molecular networks. (a) The schematic illustration of Zn deposition behaviors in TPO/Zn(OTf)_2_. (b) Statistical plots of characteristics from multiple theoretical simulation calculations for seven additives. (c) Binding energies (eV) of various additives with Zn(H_2_O)_4_^2+^. (d) Iso-surface of orbital-weighted Fukui function of three compounds. The lower parts are electrostatic potential (ESP) plots of additives. (e) H_3_O^+^ concentration distribution near the Zn (002) surface with/without TPO adsorption. Here, the reference electrolyte is named as BE (blank electrolyte, 2 M Zn(OTf)_2_). (f) Zn^2+^ concentration distribution near the Zn (002) surface with/without TPO adsorption. (g) FT-EXAFS curves in R space of TPO/Zn(OTf)_2_ electrolyte (CN: coordination numbers).

In this work, we apply topological polar surface area (TPSA) that encompasses steric hindrance and spatially distributed dipole interactions as a descriptor to engineer electrolyte additives to promote the practical development of AZIBs. Through simulations and experimental investigations on seven electrolyte additives, this study reveals that trimorpholinophosphine oxide (TPO), distinguished by its high TPSA, redistributes interfacial ions (H_3_O^+^, Zn^2+^) and significantly enhances the calendar life and electrochemical kinetics of AZIBs. The TPO-containing cells demonstrate outstanding performance by achieving a high Coulombic efficiency (CE) of 99.91% and operating steadily at 280 mA cm⁻^2^. Moreover, under intermittent aging conditions, they maintain a CE of over 95% for up to 2500 h, which far exceeds the 72-h lifespan of additive-free cells. This work demonstrates that TPO’s high topological polarity enables multisite Zn^2+^ coordination and strong adsorption on zinc anodes, which leads to extremely high calendar aging stability and high-rate performance of AZIBs (Fig. 1a).

## RESULTS AND DISCUSSION

### Molecular-bridge additive induces synergistic solvent network restructuring and interfacial ion redistribution

Of the various molecular functions explored to stabilize the zinc anode interface, organophosphorus compounds have garnered attention due to their strong coordination ability and tunable steric and electronic properties. In this work, we initiated our investigation with a series of structurally related phosphorus-based additives, which exhibit varied steric hindrance and spatially distributed dipole characteristics (Fig. S1 and Table S1). As shown in Fig. 1b, we systematically evaluated the impact of dipole moment and TPSA on battery performance. A notable positive correlation is observed between the TPSA and the average CE (Zn||Cu cells). However, no strong correlation is found between the dipole moment and the cell performance. These results indicate that the TPSA rather than merely dipole moment is a good descriptor to modulate interfacial ion transport and optimize performance of AZIBs. Subsequently, we plotted the octanol-water partition coefficient (log P) values of the seven additives against their corresponding average CE obtained in Zn||Cu cells (Fig. S2). A general trend is observed: additives with lower log P values (higher hydrophilicity) tend to deliver higher CE, with TPO exhibiting the lowest log P of −1.1 and achieving the highest CE of 99.91%, providing mechanistic insight into its superior interfacial modulation capability [30,31]. However, the correlation is not strictly linear, suggesting that overall hydrophilicity alone does not dictate performance. Instead, the spatial accessibility of polar functional groups—quantified by TPSA––emerges as a critical factor. This underscores that TPSA, often intuitively reflected by log P but providing additional steric and electronic information, serves as a more holistic descriptor for predicting additive efficacy. The balance between lipophilicity (log P) and TPSA governs additive solubility and interfacial function. Low‑log P, high‑TPSA molecules disperse well and strongly adsorb via multiple polar sites to form a water‑deficient shielding layer. Overly hydrophobic additives risk aggregation, but high localized polarity can compensate as seen for additive 2 (log P = 4.8). Beyond log P, we also evaluated the correlations between CE and other molecular descriptors, including binding energy with Zn^2+^, adsorption energy on the Zn surface, and electrostatic potential (ESP) (Figs S3–S5). While no single parameter exhibits a strictly linear correlation with CE, the combination of multiple negative ESP regions and balanced interfacial adsorption collectively accounts for the superior performance of TPO, highlighting the multidimensional nature of electrolyte additive design.

To elucidate the role of TPSA, theoretical simulations were employed to gain insight into the Zn^2^⁺ coordination environment. Firstly, we selected three additives with similar structures but different TPSA (TPO of high TPSA, PPO of medium TPSA, and CPO of low TPSA) for comparative study. Table S2 presents the binding energies of various additives complexed with Zn(H_2_O)_4_^2+^. As shown in Fig. 1c, there are two distinct binding sites of the TPO molecule, i.e. the O sites in the P=O group and the C−O−C group. While PPO and CPO merely have the O site in the P=O group. Although the binding energies at the P=O site are similar across the three additives, it is worth noting that the binding energy at the C−O−C site in TPO is approximately half that at the P=O site. This difference may facilitate Zn^2^⁺ mobility at this site, promoting easier detachment and deposition on the Zn electrode surface. Moreover, the orbital-weighted Fukui function and ESP plots of the TPO, CPO, and PPO additives are presented in Fig. 1d. It shows that the TPO exhibits more negative ESP values and a broader region of negative potentials, with a more dispersed potential distribution. These features indicate that TPO possesses multiple weak-interaction sites, which help regulate the Zn^2+^ deposition pathway via electrostatic interactions [32]. The multibinding sites and more complex ESP agree with the high TPSA of TPO, which enhance the performance of AZIBs.

Hence, we focused on TPO and further investigated the interfacial environment of TPO-adsorbed Zn electrode surface. The accumulation of protons (H_3_O^+^) at the electrode interface leads to irreversible corrosion processes, destroying the thermodynamic stability and reversibility of the electrode during both calendar-aging conditions and cycling processes [33]. Meanwhile, the concentration distribution of Zn^2+^ at the interface significantly influences the battery performance under fast-charging/discharging conditions [10]. Figure 1e reveals that the surface H_3_O^+^ number density (peak around 6.9 Å from the Zn surface) in the TPO-containing electrolyte is significantly reduced (0.0029) compared with that in the BE electrolyte (0.0066). Concurrently, the Zn^2+^ concentration profile on the TPO-modified Zn surface is markedly altered. Its peak shifts inward by ∼0.3 Å, suggesting the TPO adsorption layer forms Zn^2+^-bridged fast migration pathways (Fig. 1f). The additive’s steric hindrance also lowers the Zn^2+^ concentration at ∼12 Å, while its multibinding-site character creates a Zn^2+^-enriched microenvironment at ∼14 Å. To further gain molecular-level insight into the Zn^2+^ solvation structure in TPO-containing electrolyte from an experimental perspective, X-ray absorption fine structure (XAFS) analysis was performed. The Zn K-edge XANES spectrum of the TPO-containing electrolyte exhibits a reduced absorption edge relative to the BE electrolyte, signaling the alteration of the Zn^2+^ solvation sheath (Fig. S6) [34]. Quantitative analysis of the FT-EXAFS data further elucidates this change (Fig. 1g and Figs S7–S9). While a single Zn–O scattering path at ∼2.07 Å is observed in the BE electrolyte, two paths at ∼2.03 Å and ∼2.16 Å are resolved in the presence of TPO. These are attributed to Zn–O bonds with TPO and the remaining H_2_O, respectively (Table S3), confirming that TPO directly coordinates to Zn^2+^ and displaces water from its inner solvation sphere [35]. Additional characterization supported this interfacial modulation mechanism. Linear sweep voltammetry revealed that the TPO additive widens the electrochemical stability window and mitigates zinc foil corrosion (Fig. S10). These findings demonstrate that TPO modifies the interfacial distribution of Zn^2+^ and H_3_O^+^ via steric and adsorption-bridging effects, whereby the partial replacement of solvation water by TPO creates a water-deficient coordination environment, while the high-TPSA-driven adsorption of TPO forms a sterically hindered interfacial framework that redistributes Zn^2+^ and H_3_O^+^, suppresses side reactions, and facilitates rapid, uniform Zn^2+^ transport, providing a viable reference for further design and optimization of battery performance.

### Robust electrochemical performance via modulated interfacial kinetics

**Figure 2.**
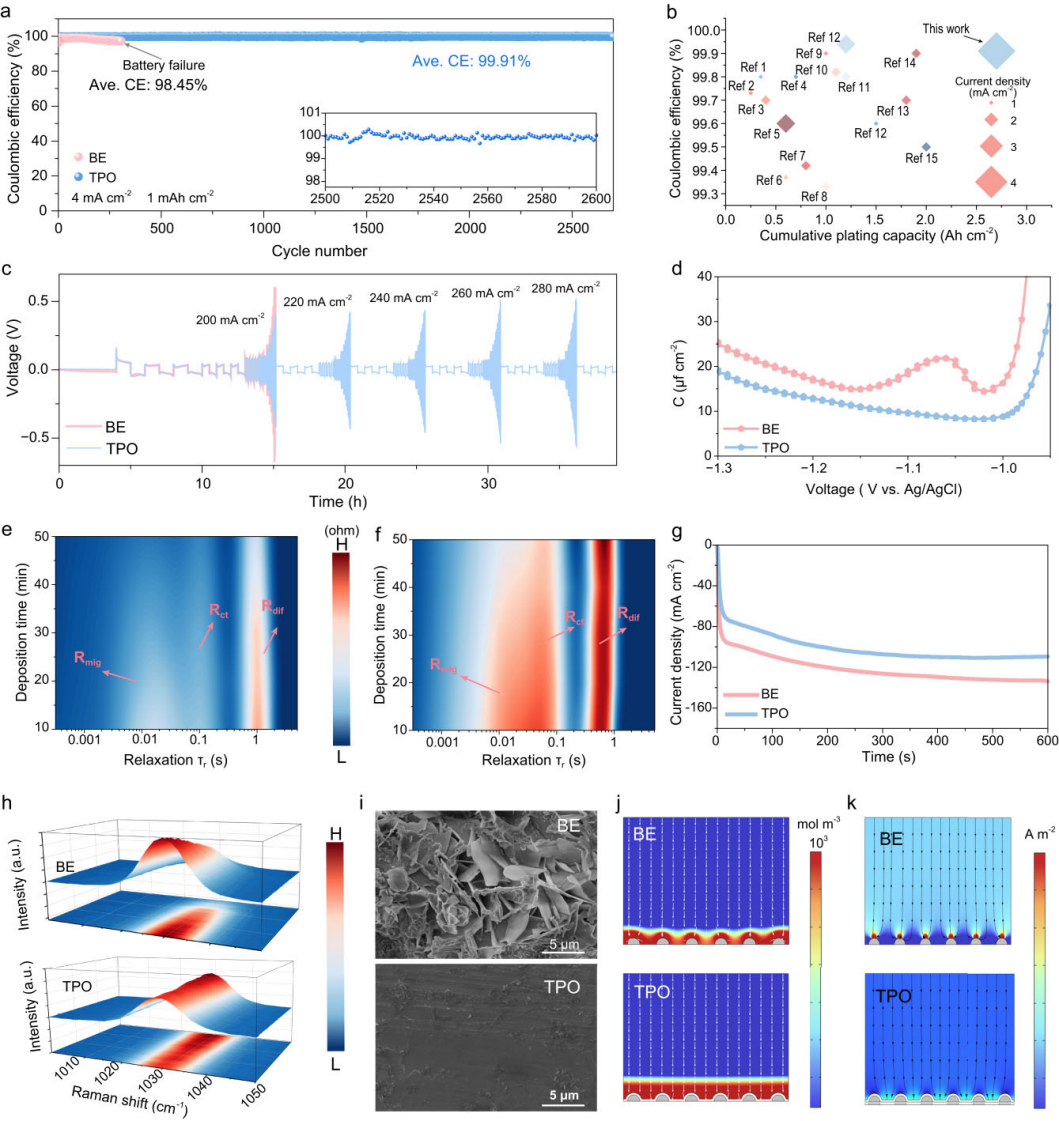
Evaluation of reversibility of zinc anodes along with kinetics investigation. (a) CE and cycling stability of Zn||Cu cells. (b) Cumulative capacity and CE comparison with reported literature. (c) Rate performance of symmetric cells at the current densities from 0.5 to 280 mA cm^−2^ in different electrolytes. (d) Differential capacitance curves for Na_2_SO_4_ solution and Na_2_SO_4_/TPO solution. The analysis of distribution of relaxation times (DRT) of operando EIS during Zn deposition in TPO/Zn(OTf)_2_ (e) and Zn(OTf)_2_ (f), respectively. (g) CA curves of Zn||Zn cells with different electrolytes at an overpotential of −150 mV. (h) *In-situ* Raman spectra of the electrode/electrolyte interface during zinc deposition in different electrolytes. (i) The SEM images of Zn anodes cycling at 2 mA cm^−2^ for 50 h in electrolytes with/without TPO. (j) The finite element analysis of Zn^2+^ flux during electrodeposition processes through COMSOL Multiphysics simulation (deposition for 60 min). (k) The COMSOL simulation results of electric field distribution on the Zn surface in different electrolytes (deposition for 60 min).

The CE of Zn||Cu half-cell configurations serves as a key indicator for quantifying the redox reversibility in AZIB systems [14]. Indeed, TPO exhibits excellent electrochemical performance in Zn(OTf)_2_ electrolytes, with an optimal concentration of 10 mM delivering the most stable cycling (Fig. S11). In Fig. 2a, the Zn||Cu cell using the TPO/Zn(OTf)_2_ electrolyte delivers a very high average CE of 99.91% for over 2700 cycles at 4 mA cm^−2^. In contrast, the cell with pure Zn(OTf)_2_ electrolyte exhibits rapid degradation and undergoes failure after only ∼300 cycles. We also compared the average CE from this work with those in other reported literature, as shown in Fig. 2b and Table S4. Clearly, adding TPO leads to a high CE and a longer cycle life, demonstrating that the TPO additive effectively improves the Zn plating/stripping reversibility.

The enhancement of high-rate cycling stability by additives was further tested through Zn||Zn cells. Notably, compared to previous studies, the cells with TPO also withstood an ultra-high current density of 280 mA cm^−2^ in gradient rate tests. Furthermore, the cells with TPO cycled stably throughout several gradient rate steps, while the symmetric cell in pure Zn(OTf)_2_ exhibited a short circuit during the initial step at 200 mA cm^−2^ (Fig. 2c and Fig. S12). It is noteworthy that during the rate test, the symmetric cell without additives failed at a depth of discharge (DOD) of only 21.5%, while the cell with additives maintained normal operation even at a DOD of 64.6% (Fig. S13). At 0.5 mA cm^−2^, the cells with TPO also showed excellent stable cycling performance (Fig. S14). Steric hindrance repels water and dipole interaction guides Zn^2+^ transport. Both effects contribute across current densities, with spatially distributed dipole interactions being critical for high-rate ion flux and steric hindrance being essential under low current density to suppress side reactions.

To probe the impact of additives on interfacial ion dynamics and electrode kinetics in Zn anodes, the differential capacitance curves of different electrolytes using a three-electrode system were tested (Zn foils were used as both working and counter electrodes). Besides, the Zn(OTf)_2_ solution was replaced with Na_2_SO_4_ solution to exclude the interference of the Faraday current. As shown in Fig. 2d, the introduction of TPO resulted in a marked reduction in capacitance, consistent with the preferred occupation of Zn surface sites by TPO and an altered inner Helmholtz plane (IHP) configuration, indicating the preferential adsorption of TPO on Zn electrodes and reconstruction of the IHP [36]. The interfacial kinetics was further compared by the distribution of relaxation times analysis, in which R_diffusion_, R_ct_, and R_mig_ denote diffusion impedance, charge transfer impedance, and migration impedance, respectively (Figs S15, S16 and Fig. 2e, f) [37]. Compared to the Zn(OTf)_2_ electrolyte, these parameters gradually decreased and became relatively smaller as Zn^2+^ plating/stripping proceeded in TPO/Zn(OTf)_2_ electrolyte, indicating enhanced interfacial dynamics and improved deposition uniformity. Chronoamperometry measurements were subsequently employed to elucidate the nucleation and growth mode of Zn on the electrode surface (Fig. 2g). The electrochemical behavior in pure Zn(OTf)_2_ electrolyte exhibited continuous current increase, attributed to non-uniform nucleation originating from two-dimensional diffusion constraints and pronounced tip effects. In contrast, adding TPO stabilized the current more quickly, acting as a bridge for three-dimensional diffusion of Zn^2+^ in the solvation-interfacial network, thereby achieving uniform deposition on the surface. Furthermore, the uniform diffusion of zinc species was further verified by *in-situ* Raman spectroscopy. *In-situ* Raman spectroscopy tracked Zn^2^⁺ variation at the Zn deposition interface via the SO_3_^2−^ signal at around 1035 cm^−1^ (Fig. 2h). Signal attenuation in the BE sample indicated severe ion heterogeneity, while TPO maintained uniform distribution by regulating ion migration.

Subsequently, the impact of additive TPO on Zn^2+^ was investigated using nuclear magnetic resonance hydrogen spectroscopy. Relative to the aqueous TPO solution, the ¹H NMR resonance of the alkane protons in TPO exhibited a distinct upfield shift in the TPO/Zn(OTf)_2_ electrolyte (Fig. S17), a phenomenon attributed to the electron-withdrawing effect arising from Zn^2+^–TPO anion coordination [38]. Furthermore, DFT calculations revealed a stronger binding affinity of TPO for the Zn (002) facet (−0.32 eV) compared to that of H_2_O (−0.08 eV), confirming the preferential adsorption of the additive on metallic zinc (Fig. S18). This indicates that TPO can promote the formation of the H_2_O-deficient protective layer on the zinc surface, thereby mitigating H_2_O adsorption-induced side reactions.

The morphological evolution of zinc electrodes during electrochemical cycling was systematically investigated through scanning electron microscopy (SEM) analysis (Fig. 2i). In the TPO/Zn(OTf)_2_ electrolyte, the morphology of zinc anodes maintained a remarkably smooth surface after 50 h of cycling, with complete suppression of dendritic growth observed across multiple length scales. Conversely, Zn surfaces cycled in Zn(OTf)_2_ electrolyte exhibited pronounced morphological degradation, manifesting as irregularly shaped blocky cluster protrusions with characteristic dendritic features. After 50 h of cycling, X-ray diffraction (XRD) of the zinc foils in the TPO/Zn(OTf)_2_ electrolyte indicated that only peaks corresponding to Zn were detected, whereas peaks corresponding to the Zn_x_OTf_y_(OH)_2x-y_·nH_2_O were observed in the zinc anode in the Zn(OTf)_2_ electrolyte (Fig. S19), indicating that the addition of TPO can continuously inhibit the side reactions. To gain further theoretical insight, finite-element analysis using COMSOL Multiphysics was performed to simulate the distribution of the electric field and ion concentration at the Zn electrode (Fig. 2j, k). The results indicate that TPO molecules at the solution and electrode interface coordinate with Zn^2+^ ions, establishing ion transport pathways that guide homogeneous Zn deposition and superior current distribution uniformity.

### Suppressing byproduct formation via *in-situ* adsorption layers

The corrosion behavior of Zn metal during the aging process was systematically investigated via immersion experiments. Following 7-day immersion in the Zn(OTf)_2_ electrolyte, irregular flake-like deposits were evident on the electrode surface via SEM (Fig. S20), whereas the Zn foil in TPO exhibited a smooth, intact morphology. To characterize the crystal phases of the aged anode surface, XRD analysis was conducted after 15 days of immersion (Fig. S21). The results demonstrated that the Zn foil soaked in pure Zn(OTf)_2_ electrolyte exhibited characteristic peaks corresponding to Zn(OH)_2_ and Zn_x_OTf_y_(OH)_2x-y_·nH_2_O on its surface, while the Zn anodes immersed in TPO/Zn(OTf)_2_ electrolyte displayed only the characteristic peaks of metallic Zn. Besides, the SEM images of the failed zinc electrode without additive clearly showed the accumulation of flake-like by-products, which may penetrate the separator (Fig. S22). Specifically, the proton-involved reaction led to an elevated interfacial OH^−^ concentration near the electrode surface, resulting in the initial formation of Zn(OH)_2_. This intermediate species subsequently reacted with triflate ions to form Zn_x_OTf_y_(OH)_2x-y_·nH_2_O [39]. Interfacial water accelerates aging via chemical corrosion during rest and desolvation polarization during cycling, necessitating an interfacial framework that simultaneously mitigates static corrosion and facilitates dynamic Zn^2+^ transport.

**Figure 3.**
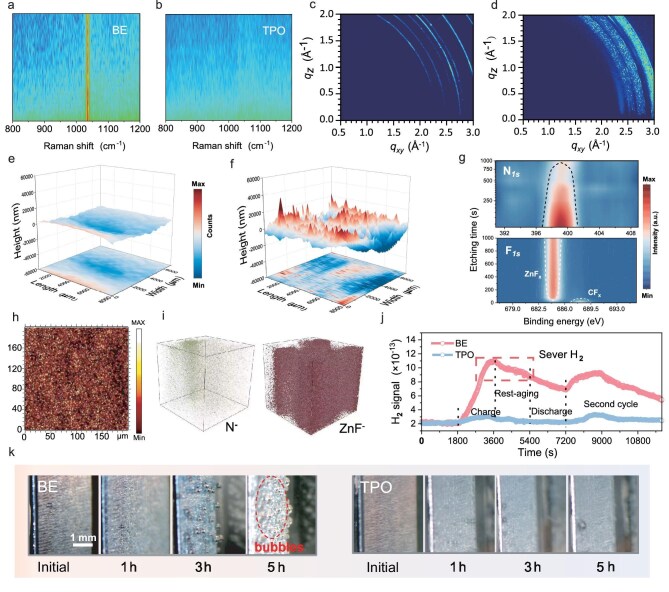
Investigations of Zn deposition behavior. Surface Raman mapping analysis of cycled Zn anodes with BE (a) and TPO (b) after cycling for 50 h at 2 mA cm^−2^, respectively. 2D GIWAXS spectrum of the cycled Zn anodes in BE (c) and TPO (d) (cycling for 100 h at 1 mA cm^−2^), respectively. Three-dimensional surface topography profiles of electrodes in different electrolytes with TPO (e) or BE (f), respectively. (g) N_1_*_s_* and F_1_*_s_* XPS depth profiles of Zn electrodes after 100 hours cycling at 1 mA cm^−2^ in the TPO/Zn(OTf)_2_ electrolyte. (h) TOF-SIMS mapping of the N^−^ composition of zinc anodes after cycling. (i) Three-dimensional spatial distribution of surface composition on zinc anode after cycling via TOF-SIMS. (j) H_2_ evolution monitored by *in-situ* DEMS. (k) *In-situ* optical micrographs of electrodes cycled in different electrolytes using the industrial optical microscope.

Fourier transform infrared analysis of the cycled Zn anode (Figs S23 and S24) confirmed the presence of C–H bending modes and characteristic vibrational signatures associated with TPO (i.e. C–O–C and C–N), consistent with the retention of adsorbed TPO at the electrode interface. The distribution of by-products was also investigated by 2D Raman spectroscopy of the zinc foils collected after recycling (Fig 3a and b). Notably, the zinc foil in Zn(OTf)_2_ revealed pronounced peaks attributed to Zn_x_OTf_y_(OH)_2x-y_·nH_2_O by-products at 1035 cm^−1^, contrasting with Zn metal derived from the TPO electrolyte, which exhibited significantly lower intensity of by-products peaks. Grazing-incidence wide-angle X-ray scattering was employed to probe the influence of electrolyte additives on the crystallographic texture and surface composition of cycled Zn anodes. As shown in Fig. 3c and d, the zinc foil cycled in TPO electrolyte exhibited lower scattering intensity in the low q_xy_ of amorphous or aggregated region compared to the zinc foil cycled in Zn(OTf)_2_ electrolyte, indicating that TPO incorporation helps to suppress the formation of surface byproducts. Furthermore, the enhanced main peak intensity observed in the high q_xy_ region (2.0–3.0 Å^−1^) suggests better preservation of the bulk crystal structure in additive-containing electrolytes [36,40]. Three-dimensional surface profilometry analyses of Zn anodes cycled in different electrolytes are depicted in Fig. 3e and f. A highly roughened surface morphology featuring irregular protrusions (vertical deviation reaching 40 000 nm) is observed for the anode cycled in Zn(OTf)_2_ electrolyte. The accumulation of disordered, flake-like by-products on the electrode surface impedes interfacial ion transport. In contrast, the anode cycled in the TPO/Zn(OTf)_2_ electrolyte retains a substantially planar surface morphology.

Subsequently, to elucidate the interfacial composition, XPS depth profiling via Ar⁺ sputtering was performed on cycled Zn anodes (Fig. 3g). N 1*s* and P 2*p* signal peaks can be detected at the Zn electrode (Fig. S25). In the core-level spectra of N 1*s*, the signals persist throughout the depth-profiling analysis, but their intensity is decreasing. This demonstrates that the additive TPO participates in forming a protective interfacial layer on the electrode surface, with predominant enrichment in the surface layer. Moreover, the signal of inorganic components ZnF_x_ is markedly intensified with increasing sputtering time. Besides, time-of-flight secondary ion mass spectrometry (TOF-SIMS) characterization with mass spectra of the ionic fragments was performed, confirming that the CN^−^ and P-containing species originate from the adsorption of the additive (Fig. S26). The inorganic components ZnF⁻ and ZnS⁻ arise from the decomposition of the electrolyte. Besides, TOF-SIMS mapping of the electrode surface indicates that P and N are primarily enriched on the surface (Fig. 3h and Figs S27, S28). Subsequently, depth profile curves obtained from TOF-SIMS analysis verify that the additive is enriched at the interfacial surface, whereas the deeper bulk region consists of inorganic species such as ZnF_x_ and ZnS_x_ (Fig. S29). The organic-rich outer layer derived from TPO adsorption limits water access while permitting anion diffusion. Trifluoromethanesulfonate anions decomposes at the Zn surface releases fluoride species, which react with Zn^2+^ to form inorganic ZnF_x_.

Furthermore, *in-situ* differential electrochemical mass spectrometry (DEMS) was added to monitor gas evolution during battery operation. As shown in Fig. 3j, intense hydrogen generation was observed during the charge and aging periods in the additive-free cell, whereas this process was significantly suppressed in the additive-containing system. In addition, to gain real-time insights into morphological evolution, an *in-situ* optical electrochemical cell was constructed for continuous monitoring of zinc anode surfaces during cycling via industrial optical microscopy (Fig. 3k). Unlike conventional approaches employing restricted observation areas (1–500 nm), this investigation implemented an expanded field of view to mitigate sampling bias inherent in localized microscopic examinations of zinc electrode surfaces. At a current density of 3 mA cm^−2^, the Zn anode cycled in the Zn(OTf)_2_ electrolyte suffered from pronounced gas bubbling and substantial by-product formation on its surface. In contrast, TPO electrolyte demonstrated a uniform surface without bubbles throughout the cycling process. The steric hindrance of TPO primarily enables the formation of a water-deficient layer that repels H_3_O^+^ and suppresses parasitic reactions, whereas its dipolar interactions enable multisite Zn^2+^ coordination and facilitate ion migration. The integration of both effects—enabled by TPO’s high TPSA—leads to a synergistic modulation of the interfacial environment, resulting in enhanced reversibility, high-rate capability, and extended calendar life.

### Superior full-cell performance and calendar aging mitigation

**Figure 4.**
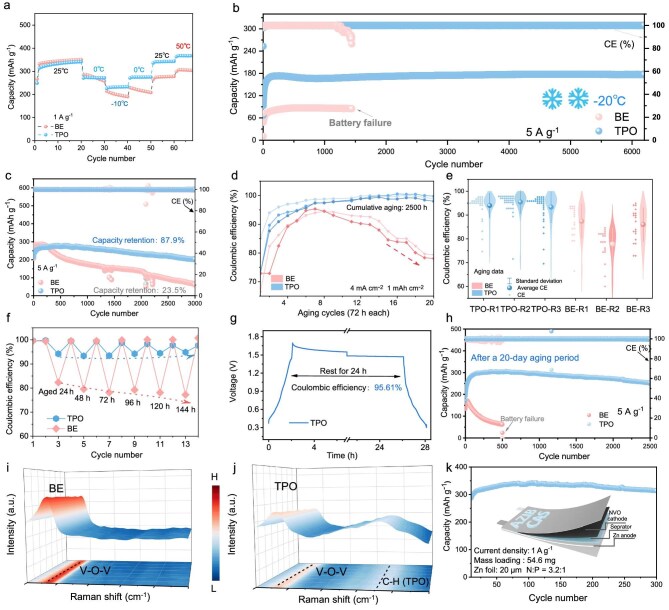
Full cell performance evaluation. (a) Comparison of capacities (full cells) in the temperature range of −10 to 50°C at 1 A g^−1^. (b) Cycling performance at a current density of 5 A g^−1^ and −20°C. (c) Cycling performance at 5 A g^−1^. (d) The statistical graph of CE during intermittent aging process in batteries. (e) Violin plot of intermittent aging CE data (R1–3: repeated data). (f) CE and cycling performance of batteries during continuous aging process. (g) Self-discharge profiles of full cells with TPO additives. (h) Cycling performance (5 A g^−1^) after 20-day aging process. *In-situ* Raman spectra of cathodes in BE (i) and TPO electrolyte (j), respectively. (k) Cycling performance of the pouch cell at 1 A g^−1^.

The synthesized NH_4_V_4_O_10_ (NVO) was employed as the cathode material to systematically investigate the effects of TPO on the electrochemical properties of the full cell. The crystallographic characterization and morphology of the synthesized NVO powder are presented in Fig. S30. As shown in Fig. S31, the CV curves of the full battery with different electrolytes are consistent, indicating that the additive does not exert any adverse effects on the redox behavior of the material. Moreover, the full battery with TPO demonstrates excellent rate performance across a current density range of 0.2–5 A g^−1^ (Fig. S32). Subsequently, the rate performance of batteries containing different electrolytes was evaluated over a wide temperature range (−10 to 50°C) at 1 A g^−1^ (Fig. 4a). The battery with TPO exhibits superior stability and higher capacity at all tested temperatures, suggesting that the TPO additive stabilizes the electrode surface and maintains rapid Zn^2+^ migration under both low- and high-temperature conditions. As shown in Fig. 4b, the full battery with TPO additive maintains a capacity of 177 mAh g^−1^ after exceeding 6000 cycles at −20°C, whereas the battery without additive fails after only 1400 cycles with a capacity of 84 mAh g^−1^. This demonstrates that the TPO additive, with its high TPSA, facilitates rapid and stable zinc ion migration even at low temperatures, enabling high-capacity and stable batteries under such conditions. Furthermore, at 60°C, the full battery with additive achieved stable cycling for over 180 cycles, with a reversible capacity of 316.94 mAh g⁻¹ and a retention of 98.8%. In contrast, the battery without additive showed a capacity of 75.86 mAh g^−1^ and a retention rate of only 20.7%, indicating that the TPO additive stabilizes the electrode interface at elevated temperatures and broadens the battery’s operational environment (Fig. S33). Subsequently, inductively coupled plasma test confirmed that the additive significantly suppressed vanadium dissolution from the full cell during cycling at 60°C (Fig. S34). Moreover, the P concentration remained nearly constant over subsequent cycles, indicating that TPO does not undergo continuous thermal consumption during high-temperature operation (Fig. S35). Subsequent variations in the interaction characteristics of different electrolytes over a wide temperature range account for the underlying mechanism of the performance enhancement. While differential scanning calorimetry measurements showed similar freezing points for both electrolytes (Fig. S36), variable-temperature ¹H NMR spectra (0–50°C) disclosed an upfield shift of the water signal in the TPO-containing electrolyte (Fig. S37). This shift, indicative of enhanced electron shielding from TPO’s weak electron-donating interactions, suggests that TPO reduces local water activity without altering bulk freezing behavior. Electrochemical impedance spectroscopy at –10°C revealed a substantially lower interfacial resistance for the TPO‑containing cell, while ionic conductivity measurements confirmed improved ion transport (Fig. S38). This temperature-dependent behavior provides critical insight into the role of TPO in modulating ion transport. At room temperature, the thermal energy is sufficient to overcome the desolvation energy barrier, and the bulk ionic conductivity is dominated by electrolyte viscosity and charge carrier concentration. However, under low-temperature conditions, the desolvation process becomes the rate-determining step for Zn^2+^ migration. TPO directly coordinates with Zn^2+^, forming a unique solvation sheath with dual binding sites. The weaker interaction at the C−O−C site facilitates easier Zn^2+^ migration. This steric-dipole synergism becomes critically beneficial at low temperatures. Consequently, the TPO-modified solvation sheath enables more facile ion transport and interfacial charge transfer specifically when thermal energy is limited, leading to the superior capacity retention and cycling stability observed at −20°C. COMSOL simulations of the system at temperatures below 0°C further corroborated the homogeneous Zn^2^⁺ transport at the interface (Fig. S39). Furthermore, the long-term durability of the batteries was assessed at varying current densities. At 0.5 A g^−1^, after 120 cycles, the full battery with TPO retained a higher capacity (352.95 mAh g^−1^) and a higher capacity retention (87.2%), whereas the battery with pure Zn(OTf)_2_ exhibited only 105.58 mAh g^−1^ and 24.4% capacity retention (Fig. S40). Additionally, even after more than 3000 cycles, the battery with TPO maintained significantly superior performance (capacity retention: 87.9%) compared to the battery without additive (23.5%) at 5 A g^−1^ (Fig. 4c and Fig. S41).

Notably, in practical energy storage applications and experimental characterization scenarios, AZIB inevitably experience varying durations of calendar aging stages during charge/discharge cycling processes—a critical precondition frequently overlooked in previous investigations on AZIB systems [17,18]. The intermittent aging performance of the half-battery was first investigated. To further elucidate the practical relevance of the intermittent aging protocol, the aging intervals were designed to mimic realistic standby scenarios: the 72-h rest period corresponds to typical weekend or short-term idle durations, while the 24-h incremental aging protocol simulates the progressive accumulation of idle periods under intermittent usage conditions—for example, daily cycling followed by overnight standby, with occasional extended idle intervals such as holidays or maintenance. This stepwise increase in aging time enables a rigorous evaluation of how the battery withstands gradually prolonged standby durations, which is highly relevant for practical applications. As illustrated in Fig. S42a and d, the battery underwent a discharge process (zinc deposition on the copper substrate) followed by 72-h aging, succeeded by a subsequent charge process (zinc stripping from copper). This procedure was followed by five routine charge-discharge cycles to simulate several usage cycles in daily life. These sequential steps constituted one complete aging cycle, which was repeated for performance evaluation. Notably, the batteries with TPO maintained a high CE above 95% for over 2500 h during the aging process, whereas the batteries without additives maintained high CE above 95% only within the 72 h of aging (Fig. 4d). Additionally, the CE of additive-modified batteries gradually increased and stabilized throughout the aging process, in contrast to the batteries without TPO which exhibited rapid decline after an initial rise. Furthermore, both the aging CE and average CE of TPO are significantly higher than the CE of BE, and this finding is further validated by replicated datasets (Fig. 4e). Subsequently, the continuous aging performance was evaluated. As shown in Fig. S42b and f, the cell experienced discharge followed by incremental aging periods (24-h increments per cycle) prior to subsequent charging. Each aging phase was followed by one complete charge/discharge step to simulate the process of continuous aging along with transient usage. The continuous aging test exhibited distinct performance trends (Fig. 4f). The cell without TPO exhibited a progressive decline in CE. In striking contrast, TPO-containing cells maintained stable CE values throughout the aging process with gradual improvement. This comparative analysis suggests that implementing multiple charge-discharge cycles during aging or prolonged storage could effectively restore electrode surface stability. Besides, compared with the BE electrolyte battery, the zinc anode in the TPO system exhibited significantly improved cycling and aging stability at a high temperature of 60°C (Fig. S43).

The long-term interfacial robustness of the full cells was further assessed by monitoring the open-circuit voltage decay over a 24 h rest period subsequent to charging (Fig. 4g and Fig. S44). A significantly enhanced CE of 95.61% was recorded for the cell employing the TPO/Zn(OTf)_2_ electrolyte, in stark contrast to the 86.63% value obtained for the additive-free Zn(OTf)_2_ electrolyte. Given that self-discharge corresponds to aging at high SOC, we further examined the SOC dependence of aging. Under high SOC (1.2 V), aging is more severe than under moderate SOC (0.8 V), yet TPO effectively delays capacity decay at high SOC and almost completely suppresses degradation at moderate SOC, demonstrating its robust protective effect across different charge states (Figs S45 and S46). Following the 20-day aging protocol, the full cells underwent constant-current (dis)charge measurements to validate the distinct performance observed in different electrolytes. As demonstrated in Fig. 4h, the battery with the TPO/Zn(OTf)_2_ electrolyte delivered exceptional stability over 2500 cycles, while the battery with Zn(OTf)_2_ electrolyte displayed poor cycling stability and accelerated failure after 500 cycles. This indicates that the TPO additive effectively suppressed interfacial corrosion, resulting in a long-term stable electrode interface and superior anti-aging performance of the battery. To further gain insight into the interface evolution of NVO, *in-situ* Raman spectra were collected (Fig. 4i and j). The Raman peak observed at 574 cm^−1^ corresponds to the V–O–V bending mode [41]. Furthermore, the detection of C–H bending vibrations characteristic of TPO verifies the presence of adsorbed additive on the post-cycling surface. Under a low N/P ratio of 2.75, the TPO-containing Zn||NVO coin cell maintained stable cycling well beyond 3000 cycles, whereas the cell without additives failed after ∼1700 cycles (Fig. S47). Furthermore, to evaluate the practicality of the TPO additive, we extended the cycling test of the pouch full cell to over 300 cycles at a low N/P ratio (Fig. 4k).

## CONCLUSIONS

In summary, through theoretical simulation and experimental studies, it is discovered that the additive TPO with high TPSA possesses multiple Zn^2+^ binding sites due to multi-dipole moments. In addition, the steric hindrance of TPO alters the solvation structure of Zn^2+^ and constructs a water-deficient interfacial layer on the zinc surface. This enables the formation of a bridging channel for Zn^2+^ transport within the electrolyte-electrode interface and induces interfacial ion redistribution, thereby significantly improving the kinetic properties and performance of batteries. Benefiting from the TPO-modified electrolyte, a superior average Zn^2+^ plating/stripping efficiency (99.91%) over 2700 cycles and excellent symmetric-cell reversibility at an ultrahigh current density of 280 mA cm^−2^ are achieved during rate tests. Surprisingly, under intermittent aging, TPO-modified cells maintain a high CE exceeding 95% for an unprecedented 2500 h during the aging process, whereas the batteries without additives maintain this state for only 72 h. Furthermore, at −20°C, the full battery with TPO maintains a capacity of 177 mAh g⁻^1^ after 6000 cycles, while the additive-free battery fails after only 1400 cycles with the capacity of 84 mAh g⁻^1^. Beyond these electrochemical achievements, this study identifies TPSA as a highly effective and integrative molecular descriptor that synergistically combines dipole interactions and steric hindrance—two critical yet often separately considered properties in additive design. Therefore, TPSA serves as a powerful design rule for interfacial regulators, paving the way to the development of durable, high-rate, aging-resistant energy storage systems.

## Supplementary Material

nwag268_Supplemental_File
