# Supplementary material for: Spatio-dipolar synergy modulated interfacial molecular bridge for calendar-aging-resistant aqueous zinc-ion batteries
Source: Natl Sci Rev. 2026 May 9;13(12):nwag268. doi: 10.1093/nsr/nwag268 (PMC13309933; doi:10.1093/nsr/nwag268)
Supplement: nwag268_Supplemental_File [file nwag268_supplemental_file.pdf]

Supplementary Data

Spatio-dipolar synergy modulated interfacial molecular bridge for calendar aging resistant aqueous zinc-ion batteries

Zimin Yang,[a,c,d,†] Yilun Sun,[a,†] Jianwei Li,[b,d] Mingqiang Wu,[a,e] Siting Deng,[a] Xinbin Nie,[b] Yifan Su,[b] Hao Tong,[a] Pengpeng Guo,[e] Jingkui Gao,[e] and Guoliang Chai[a,c,d,*]

[a]State Key Laboratory of Structural Chemistry, Fujian Institute of Research on the Structure of Matter, Chinese Academy of Sciences, Fuzhou 350002, China;

[b]Key Laboratory of Comprehensive and Highly Efficient Utilization of Salt Lake Resources, Qinghai Province Key Laboratory of Resources and Chemistry of Salt Lakes, Qinghai Institute of Salt Lakes, Chinese Academy of Sciences, Xining 810008, China;

[c]Fujian College, University of Chinese Academy of Sciences, Fuzhou 350002, China;

[d]School of Chemical Science, University of Chinese Academy of Sciences, Beijing 100049, China;

[e]College of Chemistry, Fuzhou University, Fuzhou 350108, China

***Corresponding author.** E-mail: g.chai@fjirsm.ac.cn

†Equally contributed to this work.

Materials

The ten additives were purchased from Bidepharm. $Zn(OTf)_2$ was purchased from TCI. Zinc foils (0.08 mm, 0.02 mm and 0.01 mm thickness, 99.999% purity) and Cu foils (0.05 mm thickness, 99.99% purity) were purchased from Guangdong Ares Metal Technology Co. Ltd.

Synthesis of $NH_4V_4O_{10}$ material

0.585 g of NH_4VO_3 was introduced to 30 mL of deionized water, followed by stirring for a duration of 10 minutes. Subsequently, 0.18 g of $H_2C_2O_4·2H_2O$ was slowly added, with stirring sustained until a dark blue-green solution was procured. The above solution was then transported into a 50 mL autoclave and placed in an oven at 180 °C for 6 hours. The obtained material was collected, thoroughly washed with deionized water, and then freeze-dried over a period of 2 days.

Fabrication of electrolytes

Aqueous electrolytes were formulated as follows: 2 M $Zn(OTf)_2$ (named BE), 2 M $Zn(OTf)_2$ with 10 mM TPO (TPO), 2 M $Zn(OTf)_2$ with saturated CPO (CPO), and 2 M $Zn(OTf)_2$ with 10 mM PPO (PPO). All other additives were prepared as 10 mM solutions.

Characterizations

The Nuclear Magnetic Resonance (NMR, JEOL ECZ400S, Japan), Fourier transform infrared (FTIR, Thermo Nicolet iS50), and Confocal *In-Situ* Raman Spectroscopy (LabRAM HR Evolution and Thermo Scientific DXR3) were employed to analyze the elemental and surface chemistry of the samples. The ionic conductivity was determined by a conductivity meter (DDS-11A, INESA Scientific Instrument Co. Ltd., China). Zn foils and cathodes were characterized with X-ray diffraction (XRD, MiniFlex 600, Cu-K_α radiation), Transmission electron microscope (TEM; JEOL JEM-2100F), Scanning Electron Microscopy (SEM, Hitachi SU-8010), and X-ray photoelectron spectroscopy (XPS, ESCALAB 250Xi spectrophotometer with Al-K radiation system). The *in-situ* optical microscope images were obtained on an optical microscope (XJ-906H, Shenzhen Xianjian Juye Electronics Co. Ltd., China) by using a homemade *in-situ* optical electrochemical cell. Atomic force microscopy (AFM, Bruker Dimension Icon) and Stylus Profiler (Dektak XT) were carried out to study the electrode surface. The X-ray absorption fine structure analysis (XAFS) experiment described in this paper was performed at the Shanghai Synchrotron Radiation Facility. The grazing-incidence wide-angle X-ray scattering (GIWAXS) data were obtained by the X-ray scattering instrument (Xeuss 3.0). DEMS measurements were carried out using differential electrochemical mass spectrometry (Shanghai Jingpu Ruo Technology Co., Ltd). TOF-SIMS measurements were carried out using Ion tof SIMS 5. TOF-SIMS analysis was performed using a Cs^+ sputtering gun (1 keV, 45 nA) and a Bi_3^+ analysis gun (30 keV, 1 pA), with an analysis depth of approximately 50 nm.

Electrochemical Measurements

Zn plating/stripping properties of Zn symmetric batteries and electrochemical performance of full batteries were performed with LIR2025 coin-type cells. The performance of coin-type cells was recorded on NEWARE battery-testing instrument (BTS-5V10/20/50mA, Shenzhen, China) at room temperature. The cathode was fabricated by mixing the synthesized $NH_4V_4O_{10}$(NVO) powder, super P, and polyvinylidene fluoride (PVDF) with a weight ratio of 7:2:1 using N-methyl-2-pyrrolidone (NMP) as solvent. The mixture slurry was printed on hydrophilic carbon paper and then transferred to a vacuum oven dried under 60°C for 12 h. Finally, cathodes were prepared by cutting circular discs from the dried samples. The cut electrode disks have a loading mass in the range of 2.1–2.7 mg

and a diameter of 1.2 cm. The area of the soft-pouch cells NVO electrodes and Zn anodes are measured as 9 cm^2 and 10 cm^2, respectively. The N/P ratio used in the pouch cell was approximately 3.2, and the cells were assembled under mild pressure (0.1~0.2 MPa) to ensure good electrode-electrolyte contact. Zinc foil and Whatman GF/C glass microfiber were employed as the anode and separator, respectively. Linear sweep voltammetry (LSV), cyclic voltammetry (CV), chronoamperometry (CA), and electrochemical impedance spectroscopy (EIS) were performed using an electrochemical workstation (CHI760E, Shanghai, China). The LSV test was conducted using a three-electrode system, with Ag/AgCl electrode, glass carbon electrode, and Ti anode serving as the reference, counter, and working electrodes, respectively. CV curves of Zn plating/stripping were obtained using Ti as the working electrode and Zn as the reference and counter electrodes. CA plots were carried out with Zn symmetric batteries (coin-type cells) at an overpotential of -150 mV and a duration of 400 s. EIS was measured for Zn//Zn symmetric cells with different electrolytes over a frequency range of 0.01 Hz to 100 kHz and fitted using ZView2 Analysis software. The half-cell was subjected to deposition tests at a current density of 2 mA cm^{-2} for *in-situ* Raman measurements, while the full cell was evaluated using galvanostatic charge-discharge tests at a current density of 0.5 A/g.

Computational Methods

The COMSOL model incorporates the tertiary current distribution, Nernst-Planck interface, and Deformed Geometry interface. The ion concentration follows Fick's first law of diffusion, while the electromigration is governed by the Nernst-Planck equation. A voltage difference of 0.27 V was applied between the electrodes. The initial Zn^{2+} concentration was set to 2 mol/L. The diffusion coefficient of Zn^{2+} in the electrolyte was defined as 2×10^{-9} m^2/s. The average current density across the cell was maintained at 200 A m^{-2}, with the system temperature fixed at 298 K under ambient conditions. At initialization, both models adopted a simplified bare zinc anode surface configuration featuring two electrodes of 8 μm length separated by a 6 μm gap. The protrusions on the bare zinc anode surface comprised six semicircular structures (radius = 0.3 μm) spaced 0.9 μm apart, with a 0.1 μm thick additive adsorption layer covering the negative electrode surface. Upon current application, zinc ions were driven by the electric field to diffuse through the electrolyte from the positive to the negative electrode surface, where they subsequently deposited through electrochemical reduction.

The TPSA values used in this work were obtained from the PubChem database, which are computed by using the fragment-based method implemented in the Cactvs toolkit (version 3.4.8.24). TPSA is defined as the sum of fragment contributions of polar atoms (mainly N and O and their attached hydrogens), following the method proposed by Ertl et. al.[1] This approach estimates polar surface area from molecular topology rather than from explicit 3D molecular surfaces.

The calculation of the binding energies, HOMO, and LUMO were performed with Gaussian 16 program [2]. The geometric structure of the molecules and ions were calculated with a density-functional method M06-2X [3] and a basis set aug-cc-pVTZ.[4, 5] The vibrational frequencies and thermal correction to Gibbs free energy of the molecules and ions were calculated at optimized geometry using the same level of theory. The binding energies are reported as Gibbs free energies. The electrostatic potential (ESP) was created by GaussView 6.0 from total SCF density. The isosurface of Fukui function was generated using the Multiwfn software.[6, 7] The first-principles DFT calculations of adsorption energies were performed using the Vienna *Ab initio* Simulation Package (VASP) code.[8, 9] All the calculations were carried out using Perdew−Burke−Ernzerhof exchange−correlation functional (PBE). The spin-polarized Kohn−Sham equations were solved in the plane-wave and pseudopotential framework with a cutoff of 500 eV. Gamma points were used for all the calculations. The adsorption of the TPO molecule on the Zn

(002) surface was investigated using Quantum ESPRESSO (QE). A pH value of 4 was maintained by setting the H_3O^+ concentration to 0.0001 M. To better simulate realistic experimental conditions, the 2 M concentration of $Zn(OTf)_2$ was included as electrolyte for solvation effects and enhances the reliability of adsorption energy calculations.

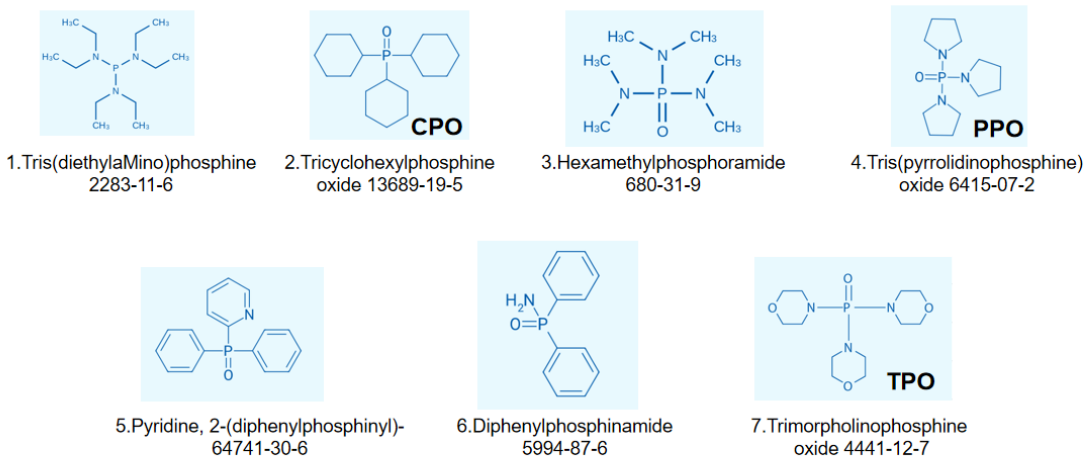

Fig. S1 The additives names, CAS numbers, and structural diagrams.

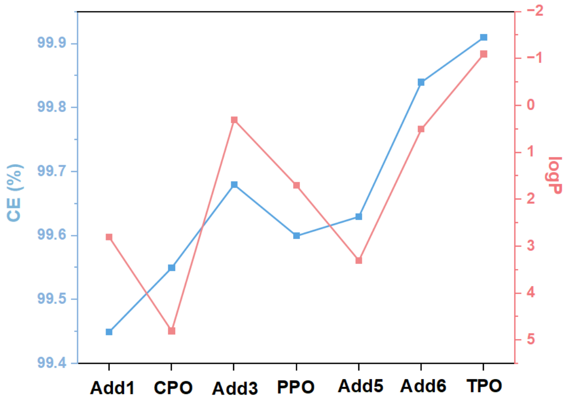

Fig. S2 Statistical plot of the octanol–water partition coefficient (logP) and CE for various additives.

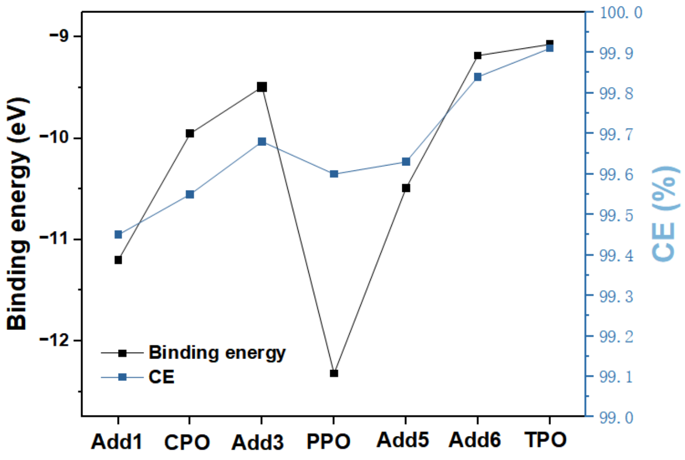

Fig. S3 Binding energies of different additives with Zn^{2+} ions and battery performance of different additives.

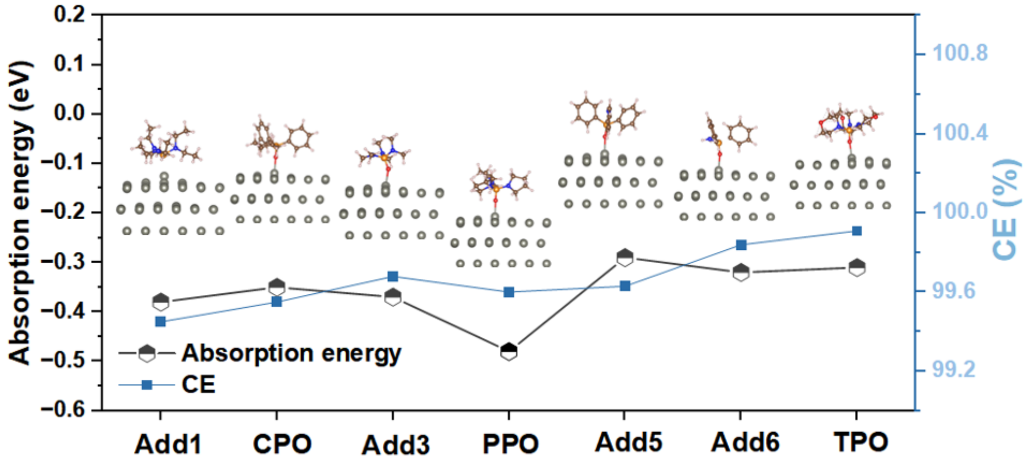

Fig. S4 Adsorption energies and battery performance of different additives on the Zn metal surface.

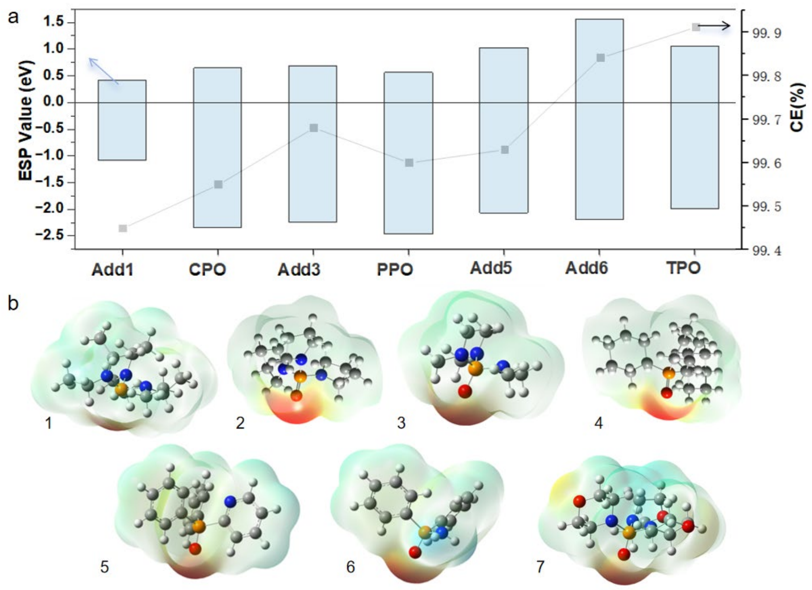

Fig. S5 (a) Electrostatic potential extrema and battery performance of different additives; (b) Electrostatic potential maps.

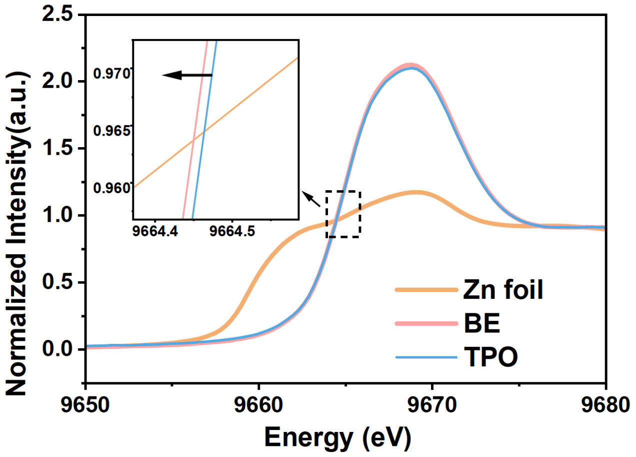

Fig. S6 Normalized X-ray absorption near-edge structure spectra of the Zn foil, $Zn(OTf)_2$, and TPO/$Zn(OTf)_2$ electrolytes.

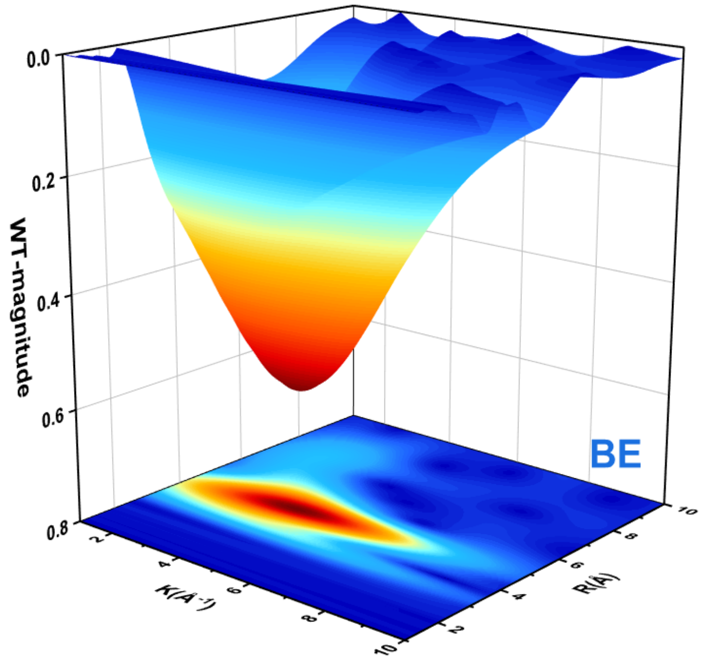

Fig. S7 Wavelet transform images of the EXAFS spectra for $Zn(OTf)_2$ electrolyte.

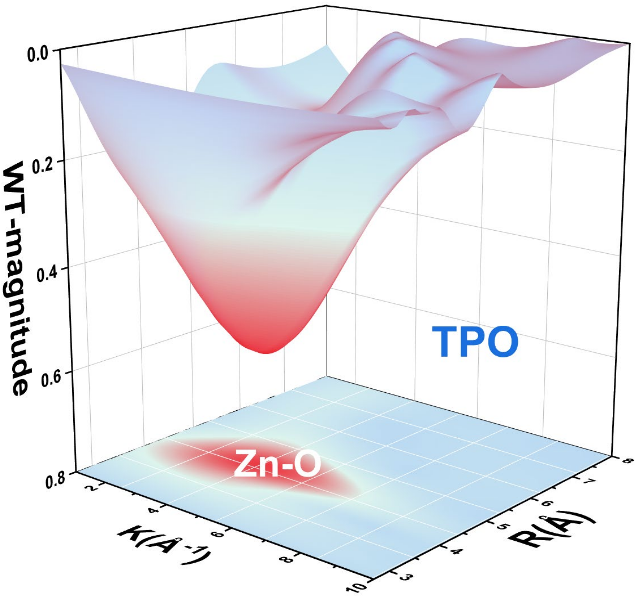

Fig. S8 Wavelet transform images of the EXAFS spectra for TPO/$Zn(OTf)_2$ electrolyte.

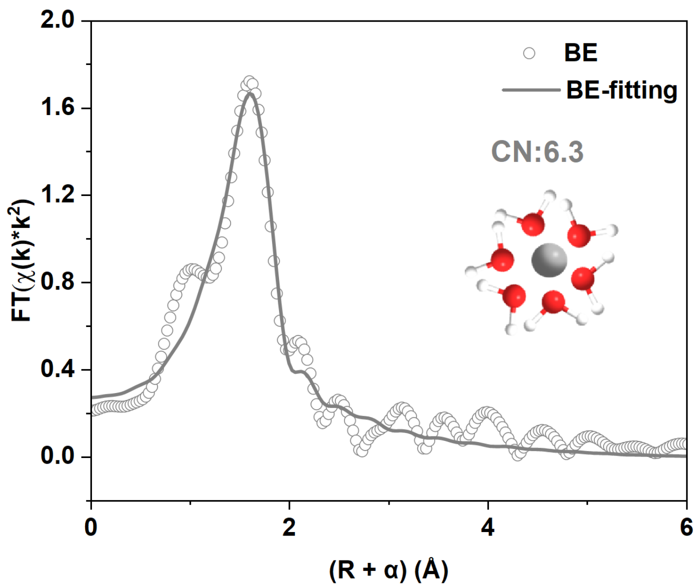

Fig. S9 FT-EXAFS fitting curves at R space of $Zn(OTf)_2$ electrolyte (CN: coordination numbers).

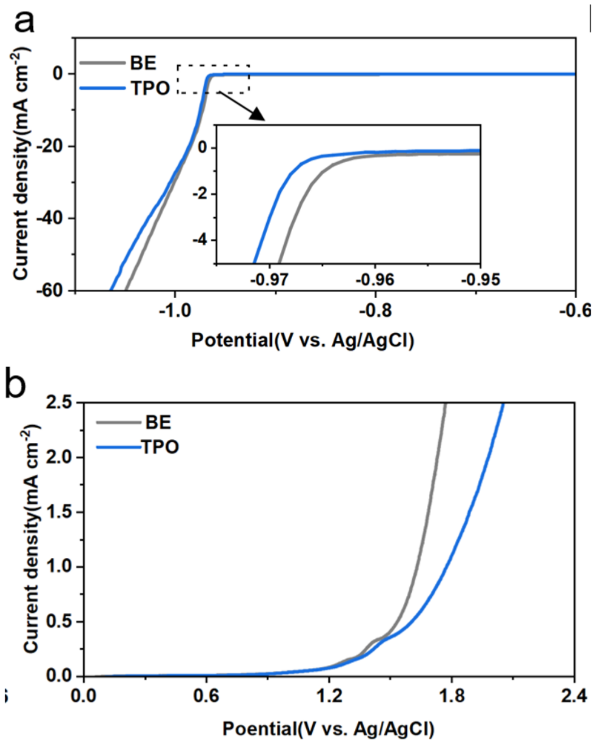

Fig. S10 Electrochemical stability window of the selected electrolytes testing on Ti electrodes.

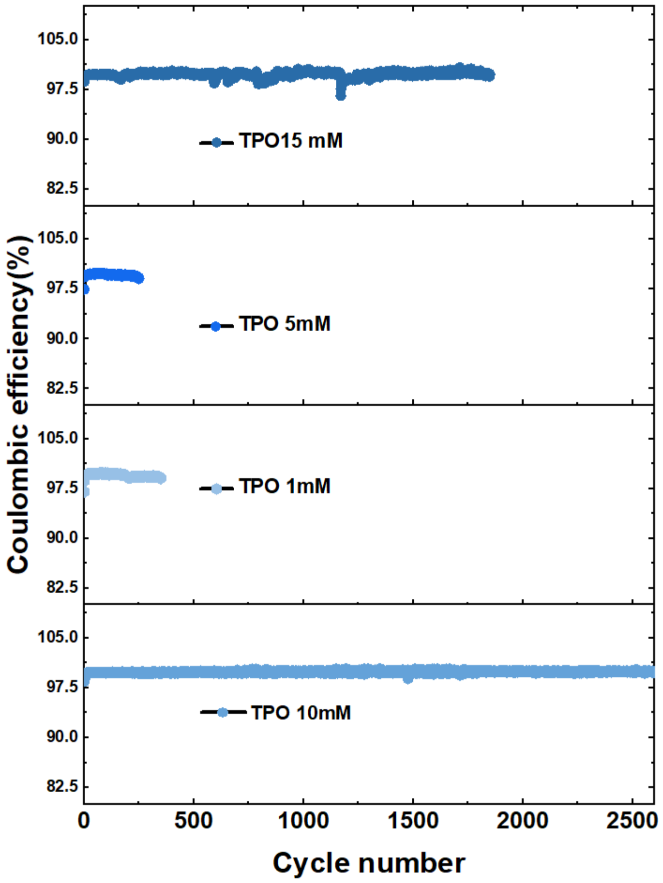

Fig. S11 CE and cycling performance of Zn//Cu cells with the different concentration additive in $Zn(OTf)_2$ electrolyte.

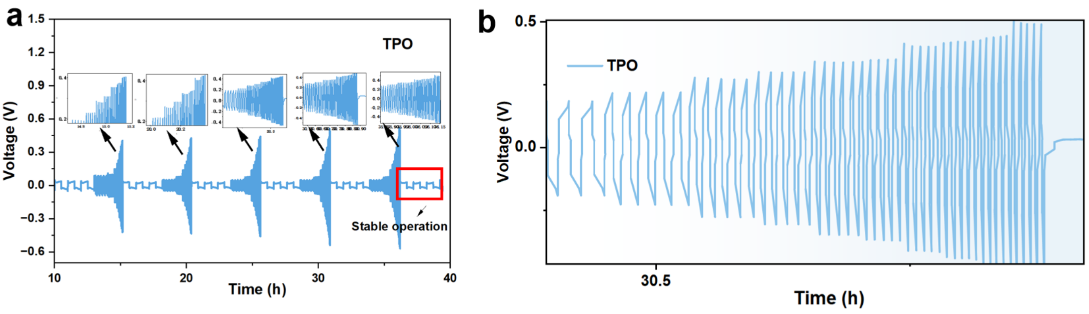

Fig. S12 (a) Voltage profiles of symmetric cells at different current densities and (b) an enlarged view of the selected region.

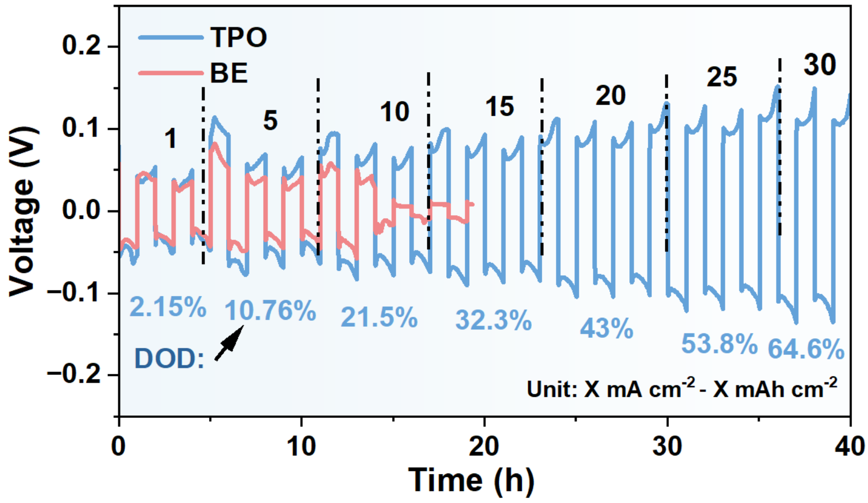

Fig. S13 Rate performance of symmetric cells at the current densities from 1 mA cm^{-2} -1 mAh cm^{-2} to 30 mA cm^{-2} - 30 mAh cm^{-2} in different electrolytes.

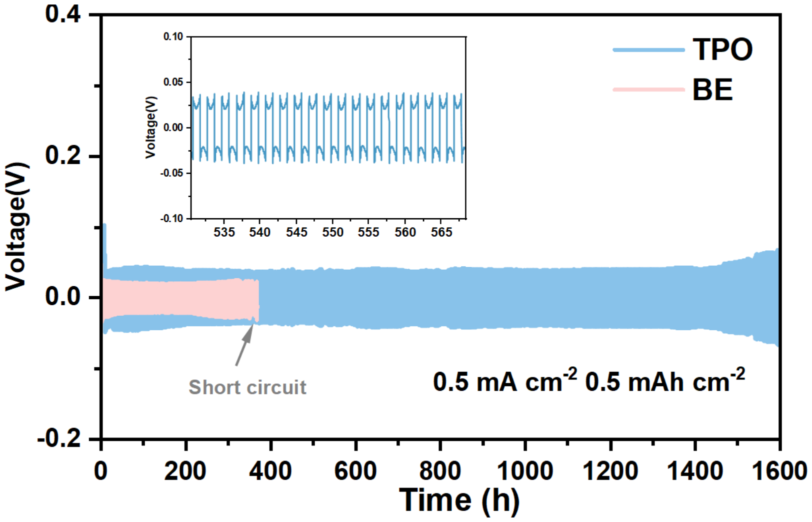

Fig. S14 Long-term galvanostatic Zn stripping/plating in symmetric cells.

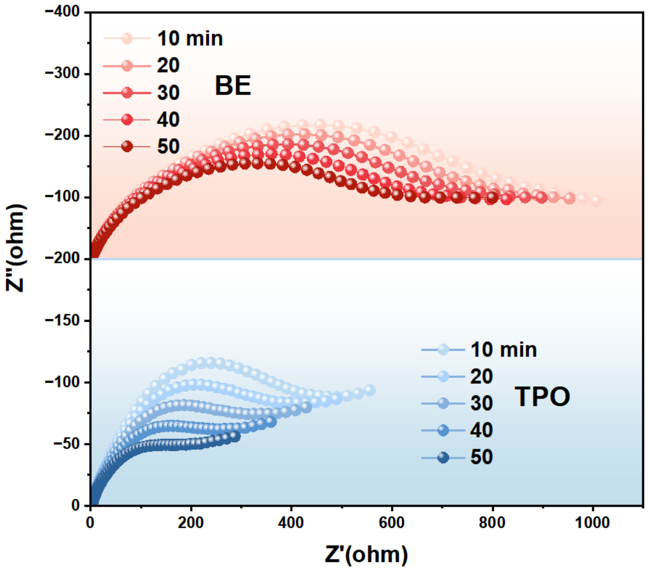

Fig. S15 Operando EIS of Nyquist plots during Zn deposition in different electrolytes.

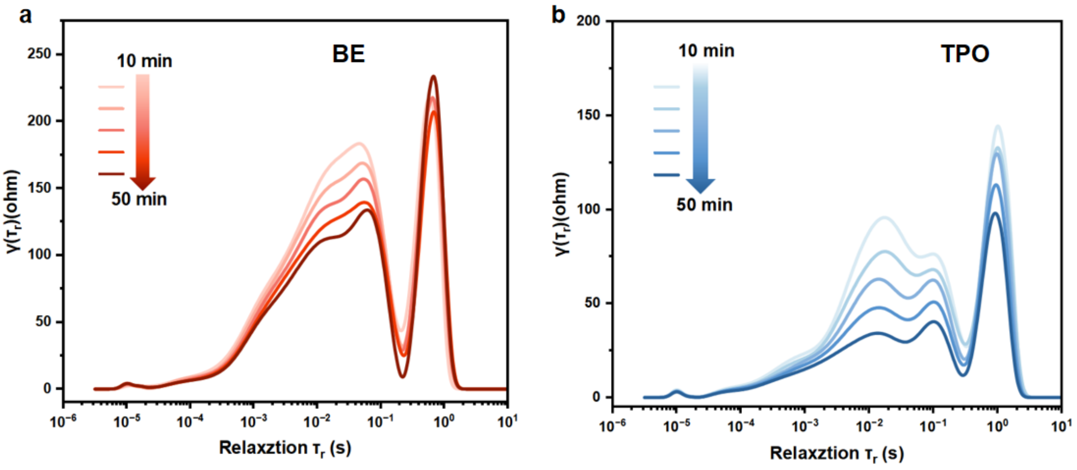

Fig. S16 DRT fitting curves for (a) BE electrolyte and (b) TPO electrolyte systems, respectively.

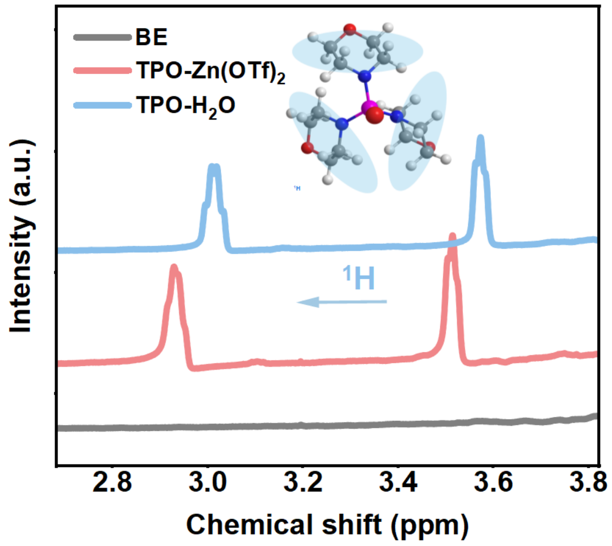

Fig. S17 ^{1}H NMR spectra of TPO/Zn(OTf)$_2$ electrolyte, aqueous solution of TPO and Zn(OTf)$_2$ electrolyte.

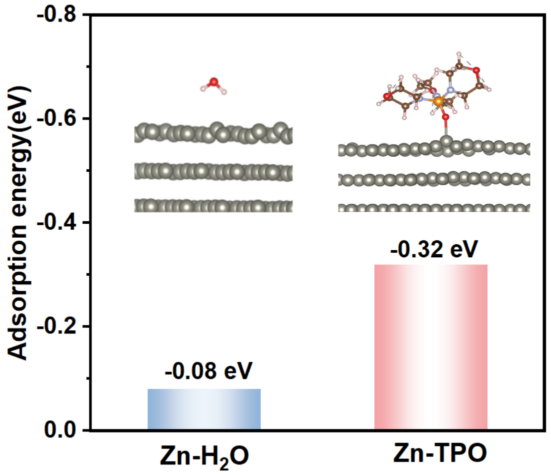

Fig. S18 The adsorption energies of the configurations with various adsorbates on the Zn (002).

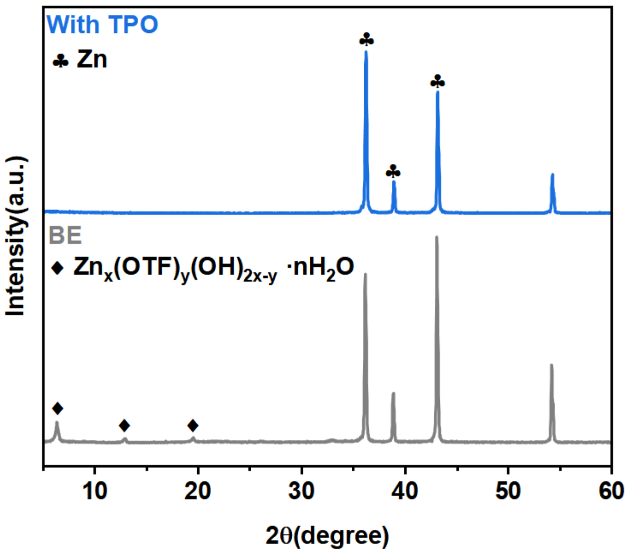

Fig. S19 XRD patterns of Zn anodes in different electrolytes after cycling for 50 hours.

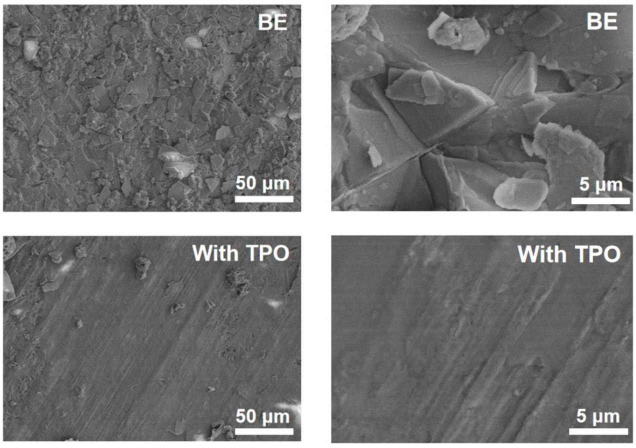

Fig. S20 The SEM images of Zn anodes in TPO/$Zn(OTf)_2$ electrolyte and $Zn(OTf)_2$ electrolyte after 7 days of immersion.

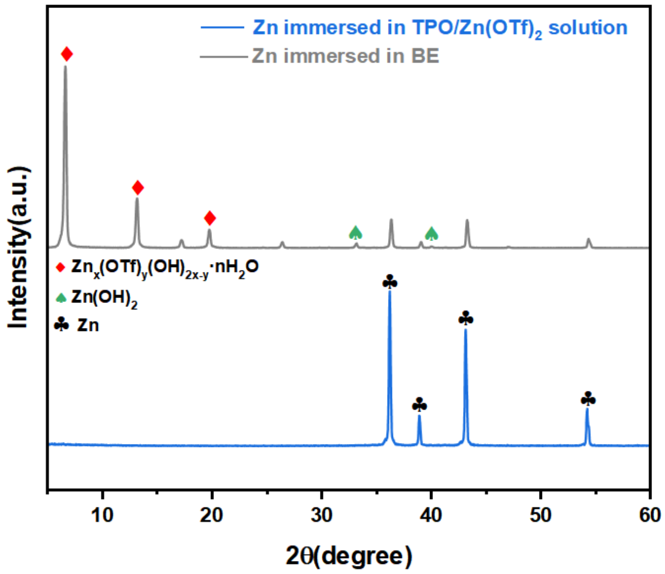

Fig. S21 XRD patterns of Zn foils after soaking in different electrolytes for 15 days.

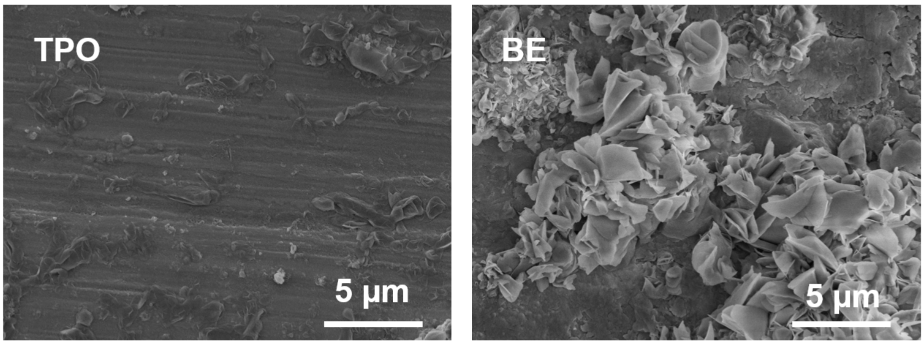

Fig. S22 Comparison of failed zinc electrode from the blank electrolyte after aging tests and the normally cycled zinc electrode from the modified electrolyte under identical conditions.

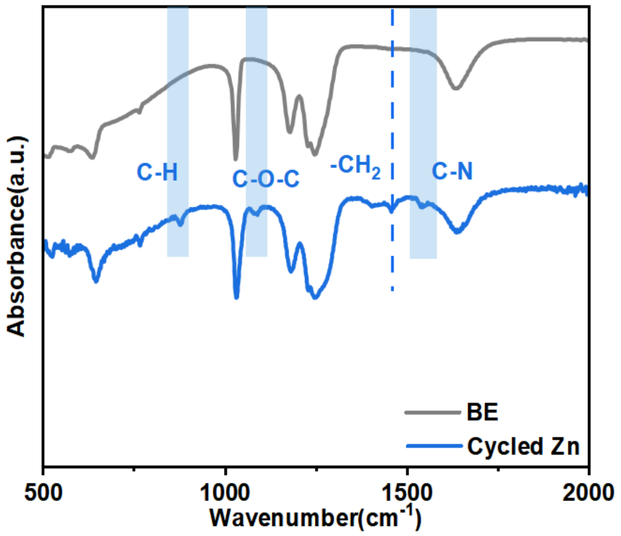

Fig. S23 FTIR spectra of the $Zn(OTf)_2$ electrolyte and cycled Zn anode in TPO/$Zn(OTf)_2$ electrolyte.

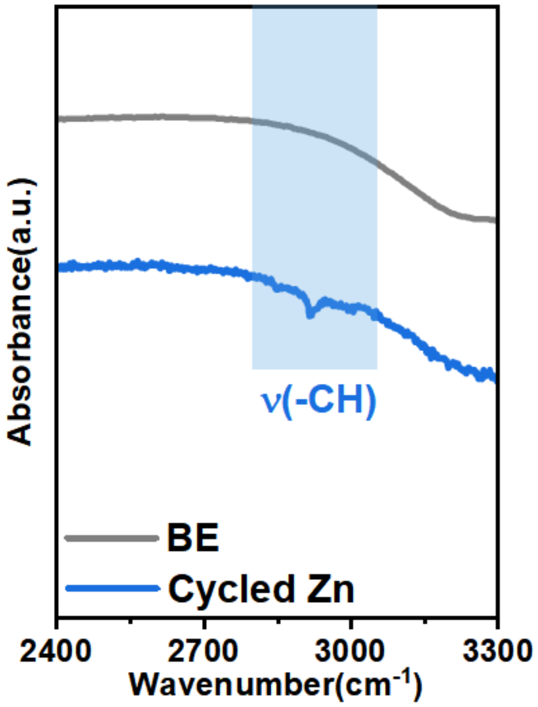

Fig. S24 FTIR spectra of the $Zn(OTf)_2$ electrolyte and cycled Zn anode in TPO/$Zn(OTf)_2$ electrolyte.

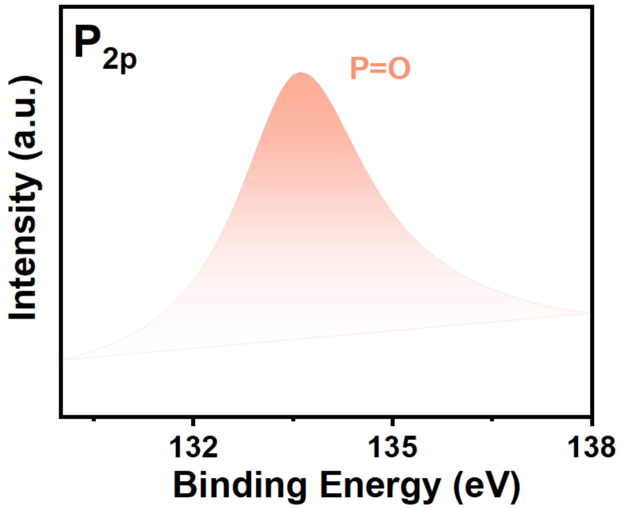

Fig. S25 The core-level P2*p* spectra of Zn foils cycled in the TPO/$Zn(OTf)_2$ electrolyte.

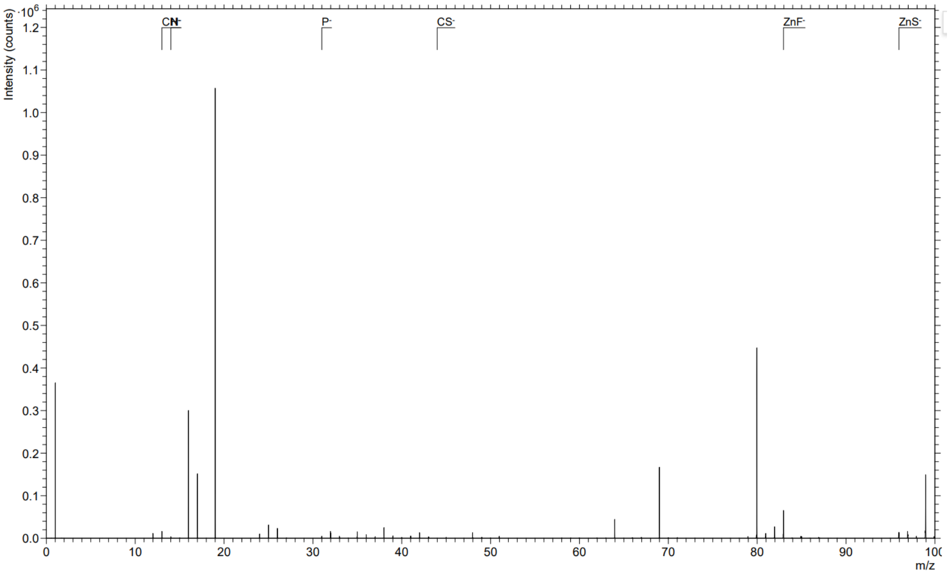

Fig. S26 Mass spectrometry analysis of ionic fragments obtained by TOF-SIMS.

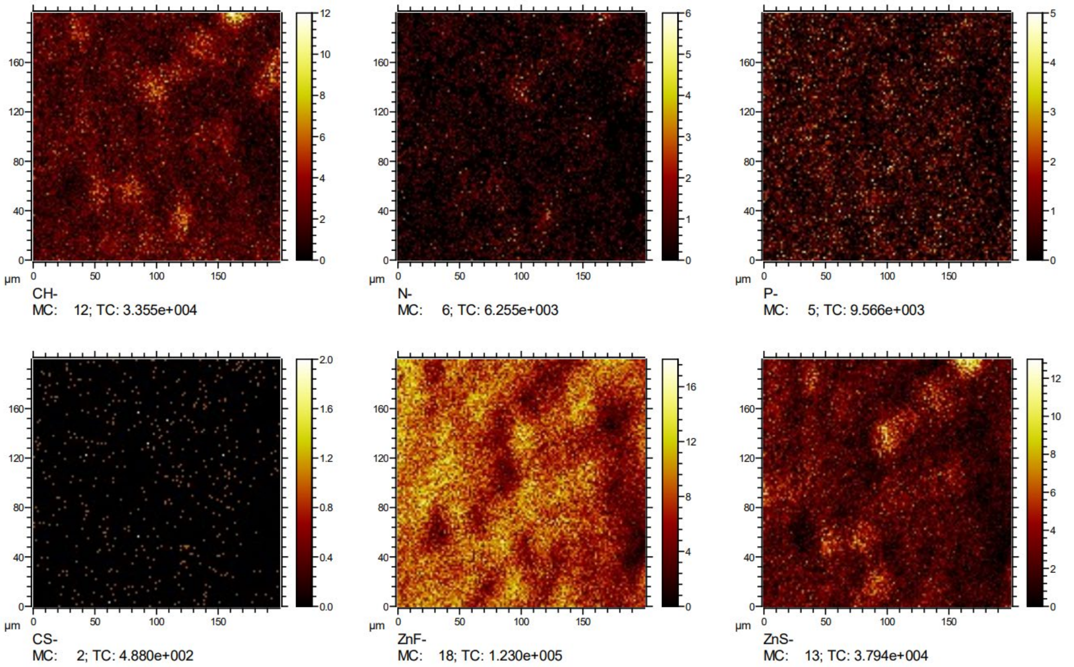

Fig. S27 TOF-SIMS mapping of the surface composition of zinc anodes after cycling.

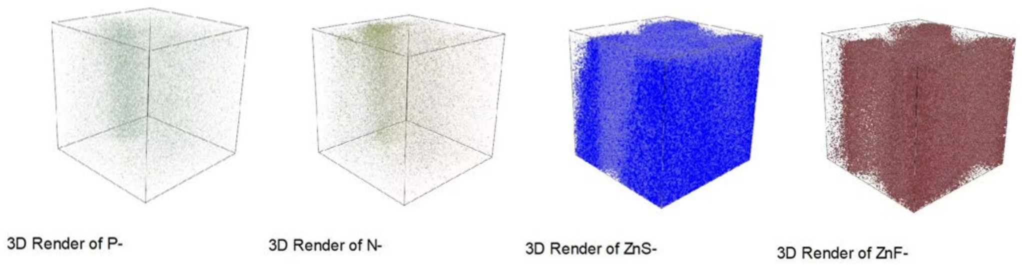

Fig. S28 Three-dimensional spatial distribution of surface composition on zinc anode after cycling via TOF-SIMS.

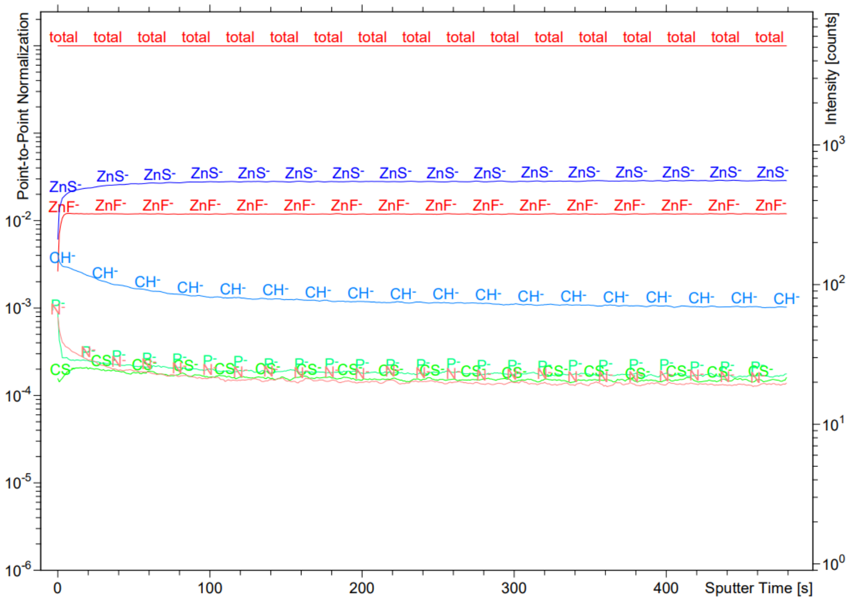

Fig. S29 Depth profile curves of mass fragments obtained by TOF-SIMS.

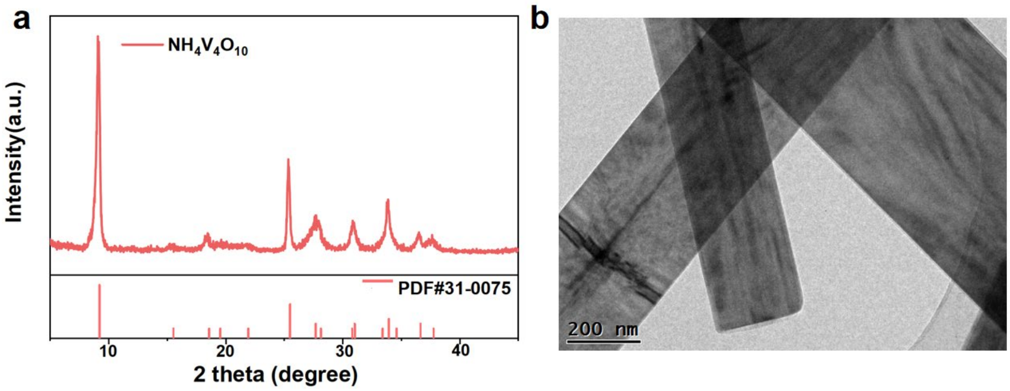

Fig. S30 The XRD pattern of the as-prepared $NH_4V_4O_{10}$ (a) and TEM image of the pristine $NH_4V_4O_{10}$ cathode (b).

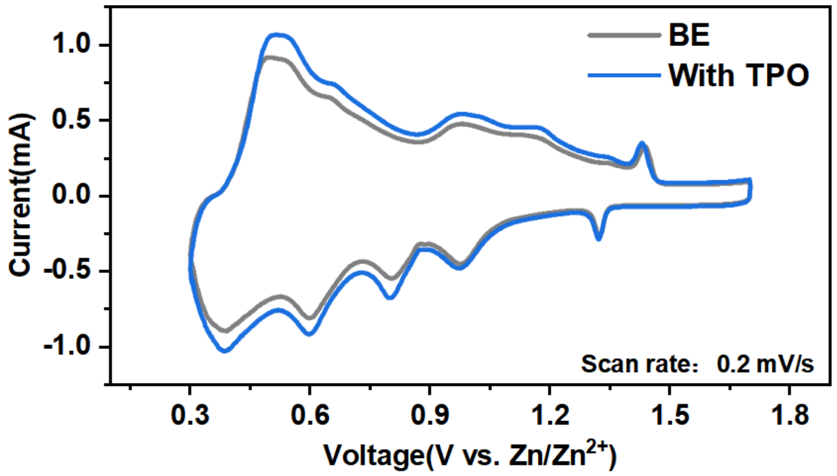

Fig. S31 Cyclic voltammetry curves of the full cells in different electrolytes.

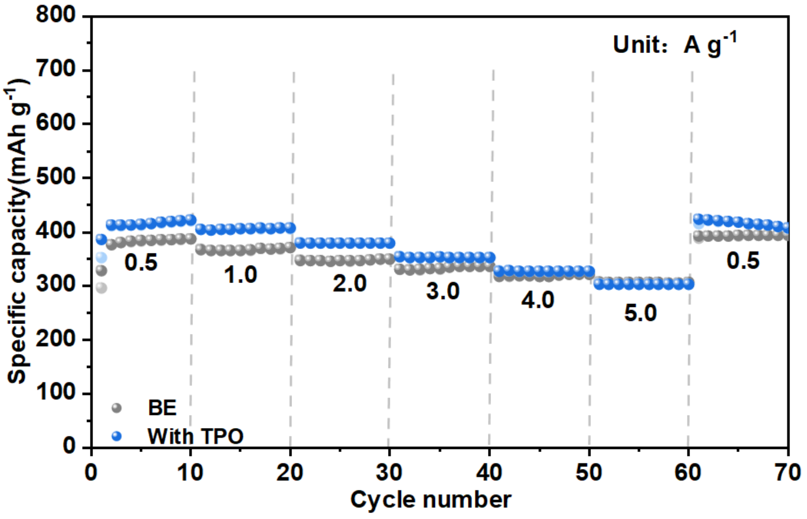

Fig. S32 Comparison of capacities of full cells at various current densities.

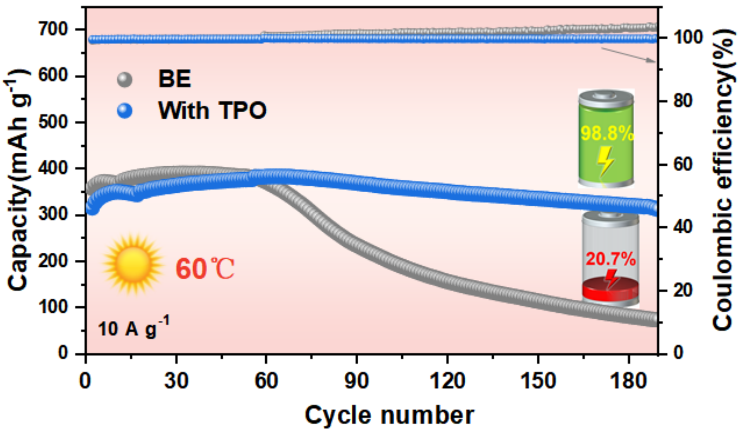

Fig. S33 Cycling performance at a current density of 5 A g^{-1} at 60°C.

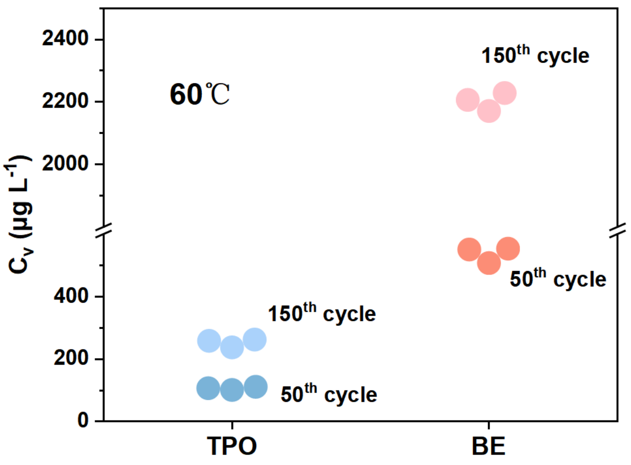

Fig. S34 V concentration in the electrolyte of full cells with different electrolytes after 50 and 150 cycles.

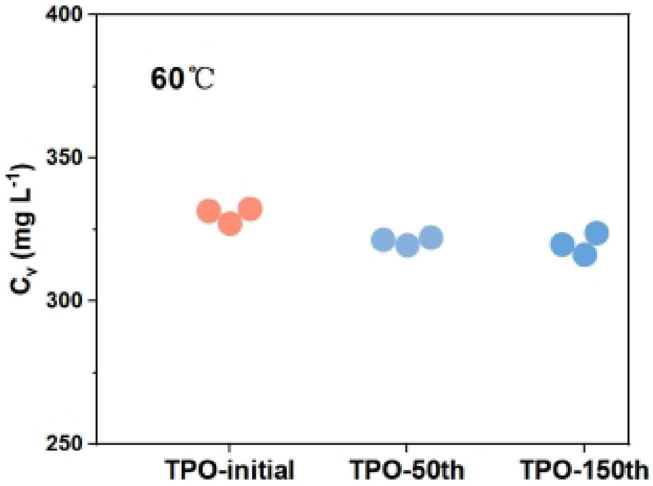

Fig. S35 P concentration in the TPO electrolyte of full cells at different state.

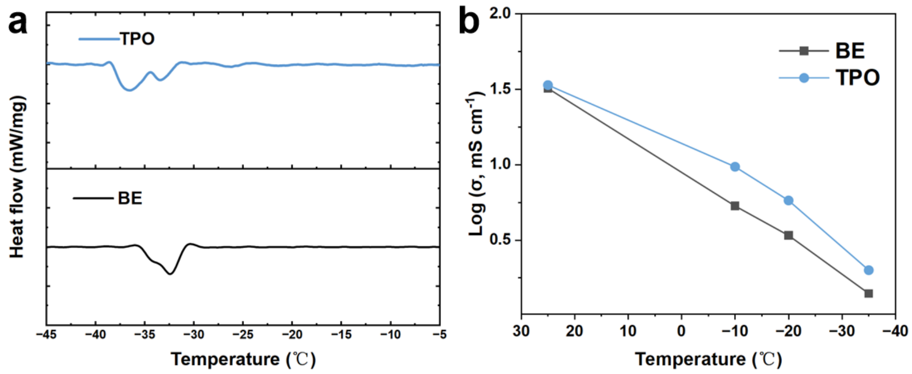

Fig. S36 (a) Low-temperature DSC curves of different electrolytes; (b) Ionic conductivity of various electrolytes at different temperatures.

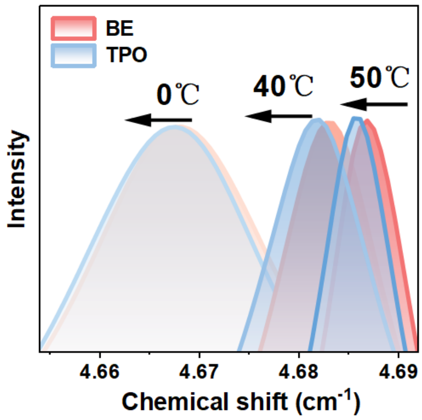

Fig. S37 ^{1}H NMR spectra of different electrolytes at various temperature.

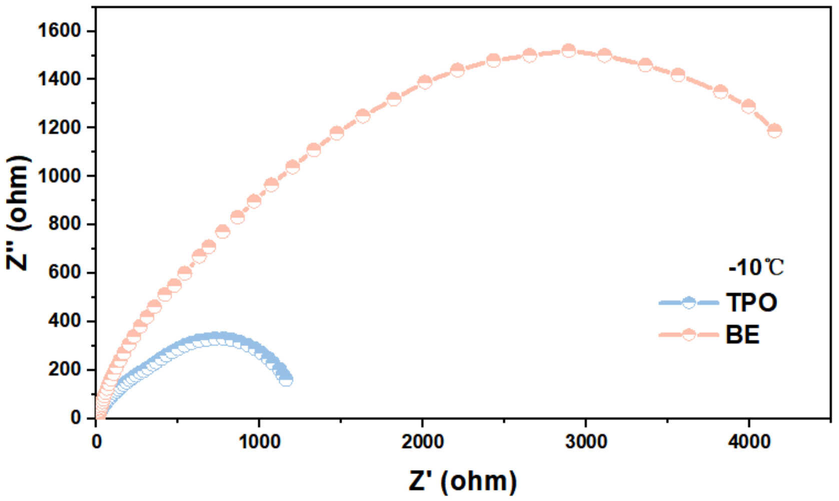

Fig. S38 Nyquist plots of Zn anodes with different electrolytes at -10℃.

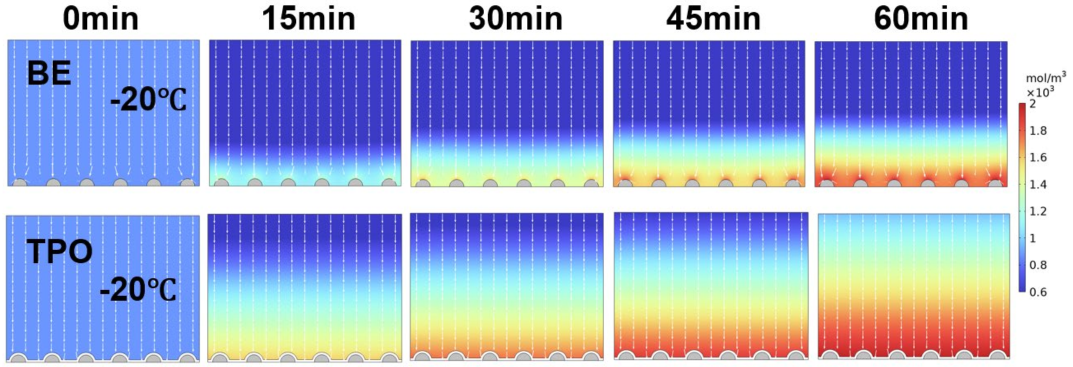

Fig. S39 The finite element analysis of Zn^{2+} flux during electrodeposition processes at -20°C through COMSOL Multiphysics simulation.

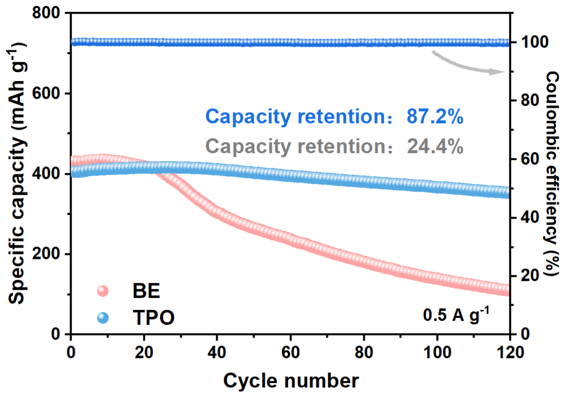

Fig. S40 Cycling performance at 0.5 A g^{-1}.

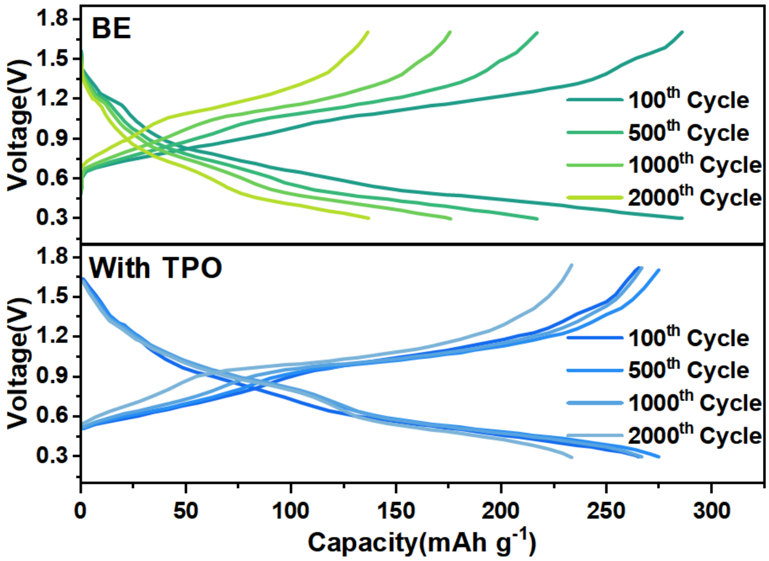

Fig. S41 The charge-discharge profiles of Zn//NVO with the BE electrolyte and TPO electrolyte.

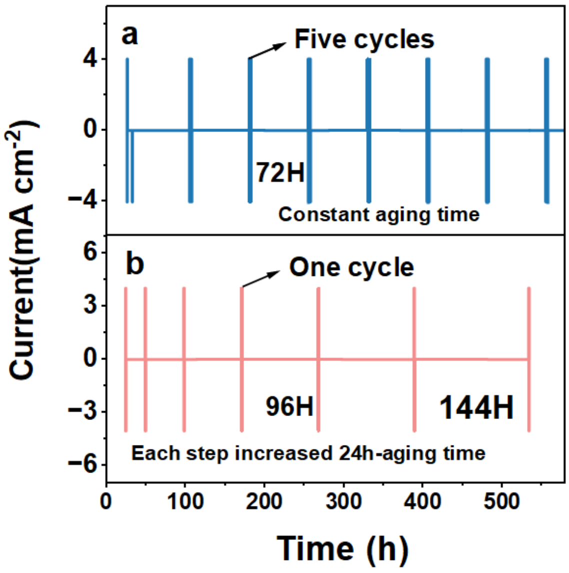

Fig. S42 Operational step schematic of intermittent aging (a) and continuous aging procedures (b).

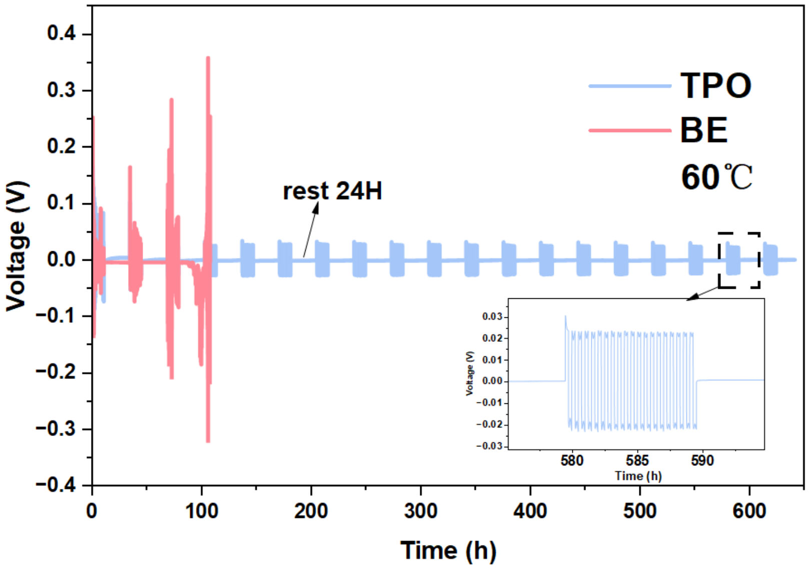

Fig. S43 Calendar life and long-term cycling performance of the Zn anodes with different electrolytes at 60 °C.

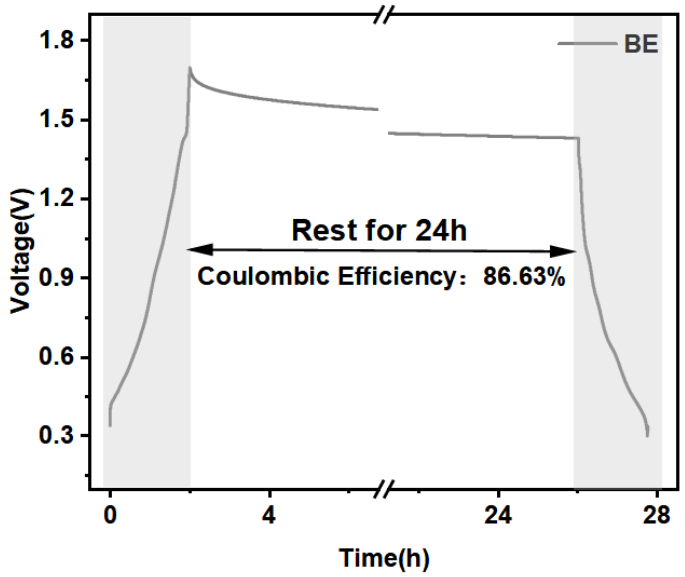

Fig. S44 Self-discharge curves of full cells in electrolytes without TPO additive.

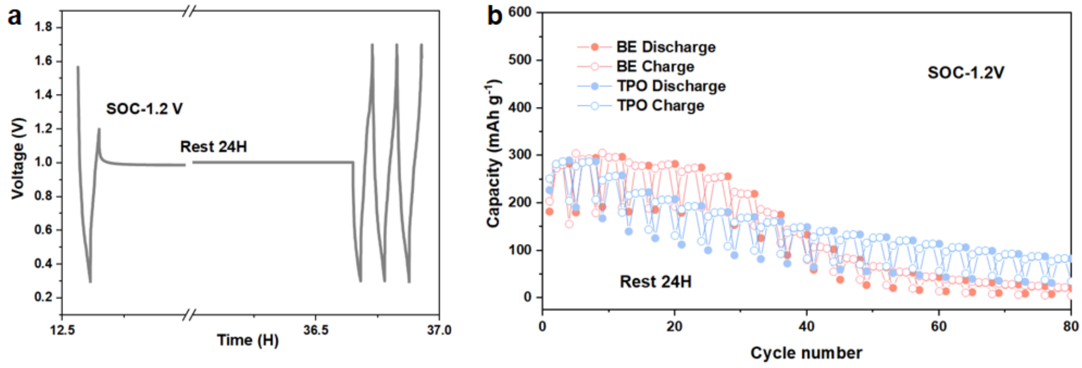

Fig. S45 (a) Calendar aging and long-cycling protocol at SOC of 1.2 V. (b) Specific capacity plot of full cell aging-long cycle at SOC of 1.2 V.

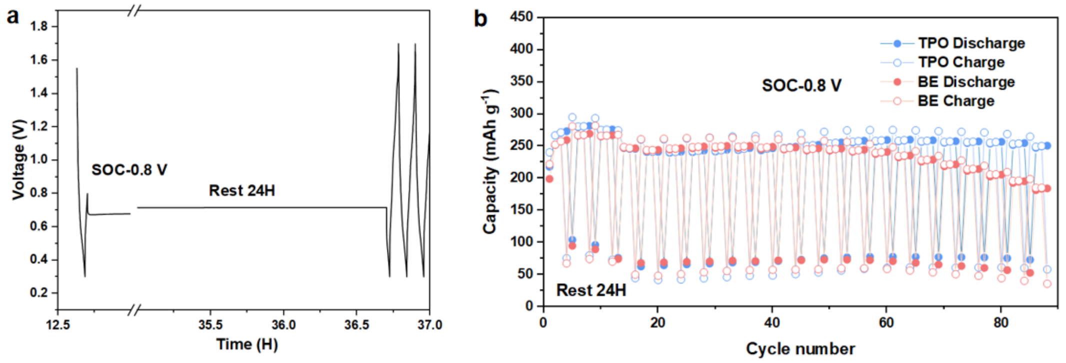

Fig. S46 (a) Calendar aging and long-cycling protocol at SOC of 0.8 V. (b) Specific capacity plot of full cell aging-long cycle at SOC of 0.8V.

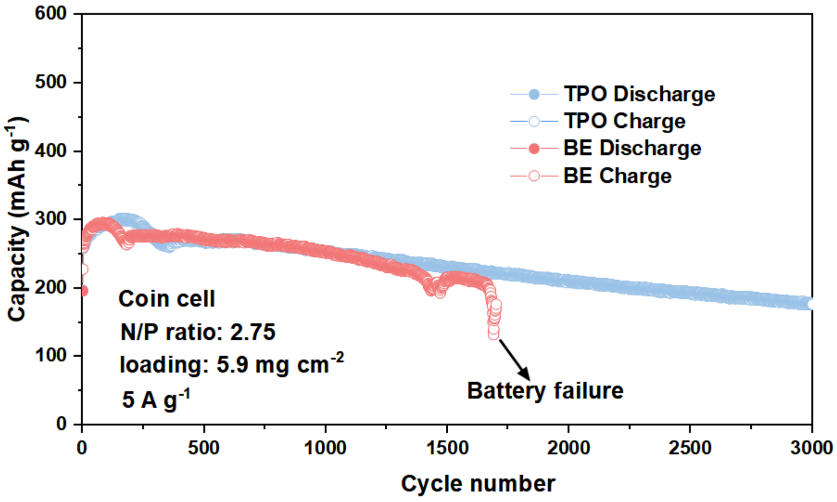

Fig. S47 Cycling performance of the coin full cells at 5 A g^{-1}.

Table S1. Fitting results for symmetric cells at different temperatures.

Additive	TPSA ($Å^2$)	Dipolemoment	Average CE (%)	CAS number	logP
1.Tris(diethylaMino)phosphine	9.7	0.943	99.45	2283-11-6	2.8
**2.Tricyclohexylphosphine oxide (CPO)**	17.1	3.904	99.55	13689-19-5	4.8
3.Hexamethylphosphoramide	26.8	3.920	99.68	680-31-9	0.3
**4.Tris(pyrrolidinophosphine) oxide (PPO)**	26.8	4.43	99.6	6415-7-2	1.7
5.Pyridine, 2-(diphenylphosphinyl)-	30	3.545	99.63	64741-30-6	3.3
6.Diphenylphosphinamide	43.1	3.559	99.84	5994-87-6	0.5
**7.Trimorpholinophosphine oxide (TPO)**	**54.5**	**2.588**	**99.91**	**4441-12-7**	**-1.1**

Table S2. Binding energies (eV) of various additives with $Zn(H_2O)_4^{2+}$, calculated with the M06-2X/cc-pVTZ theoretical level.

Additive (binding site)	Binding Energies
TPO (P=O)	-2.92
TPO (C-O-C)	-1.43
CPO (P=O)	-3.26
PPO (P=O)	-3.56

Table S3. Fitting results of EXAFS of different samples

Sample	Path	CN	R(Å)	$\sigma^2(10^{-3}Å^2)$	$\Delta E_0(eV)$	R factor
BE	Zn-O	6.3	2.07	7.8	0.357	0.024
TPO	Zn-O (additive)	3.1 (2~3)	2.03	3.0	2.19	0.016
	Zn-O (H_2O)	3.3 (3~4)	2.16	7.0	2.19	0.016

CN: coordination numbers; R: bond distance; σ^2: Debye-Waller factors; ΔE_0: the inner potential correction; R factor: goodness of fit. S_0^2 was set to 0.96. The data ranges are presented as $3 \le k \le 12.269$ Å^{-1}, $1 \le R \le 2.6$ Å for BE; $3 \le k \le 12.27$ Å^{-1}, $1 \le R \le 2.6$ Å for TPO. Error bounds that characterize the structural parameters obtained by EXAFS spectroscopy were estimated as CN ± 20%, σ^2 ± 20%, R ± 0.03 Å. To ensure the accuracy of our fitting, we focused on the main coordination peaks (i.e., Zn–O paths at ~2.03 Å and ~2.16 Å).

Table S4. Performance of Zn//Cu symmetric cells using TPO/$Zn(OTf)_2$ electrolyte compared with other reported literatures.

No.	Current density (mA cm^{-2})	Coulombic efficiency	Cumulative plated capacity (Ah cm^{-2})	Article information
1	0.5	99.8	0.35	ACS Energy Lett. 2023, 8, 5, 2086–2096
2	0.5	99.73	0.25	Adv. Funct. Mater. 2023, 2214538
3	1	99.7	0.4	Angew. Chem. Int. Ed. 2024, 63, e202409957.
4	0.5	99.8	0.7	Angew. Chem. Int. Ed. 2023, 62, e202311268
5	2	99.6	0.6	Angew. Chem. Int. Ed. 2023, 62, e202303557
6	0.5	99.37	0.6	Angew. Chem. Int. Ed. 2024, 63, e202410422.
7	1	99.42	0.8	Adv. Mater. 2023, 2307219
8	1	99.33	1	Adv. Funct. Mater. 2024, 35, 2410855.
9	0.5	99.9	1	Angew. Chem. Int. Ed. 2024, 63, e202407909.
10	1	99.82	1.1	Angew. Chem. Int. Ed. 2024, 63, e202412173.
11	1	99.8	1.2	Adv. Mater. 2024, 36, 2406071.
12	0.5	99.6	1.5	ACS Energy Letters **2024** 9 (10), 4985-4993
13	1	99.7	1.8	10.1016/j.cej.2024.152789.
14	1	99.9	1.9	Nat Commun 2023, 14, 5443
15	1	99.5	2	Nat Commun 2023, 14, 2720
**This Work**	**4**	**99.91**	**2.7**	**This work**

References

1. Ertl P, Rohde B, Selzer P. Fast Calculation of Molecular Polar Surface Area as a Sum of Fragment-Based Contributions and Its Application to the Prediction of Drug Transport Properties. *J Med Chem* 2000; **43**: 3714-7.
2. Risch, M. J. et al. Gaussian16 Revision B.01. 2016.
3. Zhao Y, Truhlar DG. The M06 suite of density functionals for main group thermochemistry, thermochemical kinetics, noncovalent interactions, excited states, and transition elements: two new functionals and systematic testing of four M06-class functionals and 12 other functionals. *Theor Chem Acc* 2008; **120**: 215-41.
4. Dunning TH, Jr. Gaussian basis sets for use in correlated molecular calculations. I. The atoms boron through neon and hydrogen. *The Journal of Chemical Physics* 1989; **90**: 1007-23.
5. Kendall RA, Dunning TH, Jr., Harrison RJ. Electron affinities of the first-row atoms revisited. Systematic basis sets and wave functions. *The Journal of Chemical Physics* 1992; **96**: 6796-806.
6. Lu T, Chen F. Multiwfn: A multifunctional wavefunction analyzer. *J Comput Chem* 2012; **33**: 580-92.
7. Lu T. A comprehensive electron wavefunction analysis toolbox for chemists, Multiwfn. *The Journal of Chemical Physics* 2024; **161**.
8. Kresse G, Hafner J. Ab initio molecular-dynamics simulation of the liquid-metal--amorphous-semiconductor transition in germanium. *Physical Review B* 1994; **49**: 14251-69.
9. Kresse G, Furthmüller J. Efficiency of ab-initio total energy calculations for metals and semiconductors using a plane-wave basis set. *Comput Mater Sci* 1996; **6**: 15-50.
